# Overcoming IGF1R-mediated resistance to oncolytic HSV1 and radiotherapy via triple combination therapy

**DOI:** 10.1038/s41419-026-09090-5

**Published:** 2026-07-17

**Authors:** Alexandra A. Miller, Min-Hye Noh, Jin Muk Kang, Jiyeon Kim, Lily Nguyen, Amanda S. Kouaho, Grace Nguyen, Minxin Huang, Stephanie M. Bean, Joshua Davis, Matthew P. Mullarkey, Sunil Krishnan, Zhongming Zhao, E. Antonio Chiocca, Tae Jin Lee, Ji Young Yoo

**Affiliations:** 1 Department of Neurosurgery, McGovern Medical School, The University of Texas Health Science Center at Houston, Houston, TX USA; 2 The University of Texas MD Anderson Cancer Center UTHealth Houston Graduate School of Biomedical Sciences, Houston, TX USA; 3 Department of Pediatric Hematology & Oncology, University Hospitals Cleveland Medical Center, Cleveland, OH USA; 4 Department of Pathology, University of Texas Southwestern Medical Center, Dallas, TX USA; 5 Molecular and Cellular Pharmacology Program, Department of Pharmacological Sciences, Renaissance School of Medicine at Stony Brook University, Stony Brook, NY USA; 6 Center for Precision Health, McWilliams School of Biomedical Informatics, The University of Texas Health Science Center at Houston, Houston, TX USA; 7 Harvey W. Cushing Neuro-oncology Laboratories, Department of Neurosurgery, Brigham and Women’s Hospital and Harvard Medical School, Boston, MA USA

**Keywords:** Cancer immunotherapy, Radiotherapy, Cancer therapeutic resistance, Cancer microenvironment, Breast cancer

## Abstract

FDA-approved oncolytic herpes simplex virus-1 (oHSV) therapy has emerged as a promising viro-immunotherapy for solid tumors. However, tumor- and tumor microenvironment (TME)-associated adaptations following viral treatment, such as feedback immune suppression, neoangiogenesis, and enhanced tumor aggressiveness, often hinder complete tumor eradication. A deeper understanding of the molecular mechanisms underlying resistance to oHSV is crucial to enhancing its clinical impact. We recently discovered that oHSV induces Insulin-like growth factor 2 (IGF2) secretion, shaping an immunosuppressive TME. Similarly, radiotherapy (RTx) activates the IGF1/IGF1R and YAP1 signaling pathways, further promoting therapeutic resistance. In this study, we investigated how oHSV-induced Insulin-like growth factor 1 receptor (IGF1R) signaling drives feedback pro-survival and proliferative pathways in tumor cells and evaluated the therapeutic potential of combining IGF1R blockade with oHSV and RTx. We first demonstrated that oHSV activates IGF1R signaling in vitro and in vivo, promoting tumor proliferation. While IGF1R-targeted monotherapies have shown limited cytotoxicity, its combination with oHSV led to a modest but significant increase in cytotoxicity across tested breast cancer (BC) and primary glioblastoma (GBM) cells in vitro and in vivo xenograft models. Furthermore, we observed that co-treatment with oHSV and RTx robustly activated both IGF1R and YAP1 in resistant cells, revealing the IGF1R/YAP1 axis as a key mediator of resistance to dual oHSV and RTx therapy. Notably, the triple combination of oHSV, RTx, and IGF1R blockade yielded synergistic anti-tumor effects, abolished YAP1 expression and nuclear localization, and significantly enhanced survival in orthotopic BC and GBM models. Collectively, these findings identify the IGF1R/YAP1 axis as a critical driver of resistance to oHSV and RTx and provide a strong rationale for the clinical evaluation of this triple-combination strategy to enhance therapeutic efficacy in patients with BC and GBM.

**Figure:**
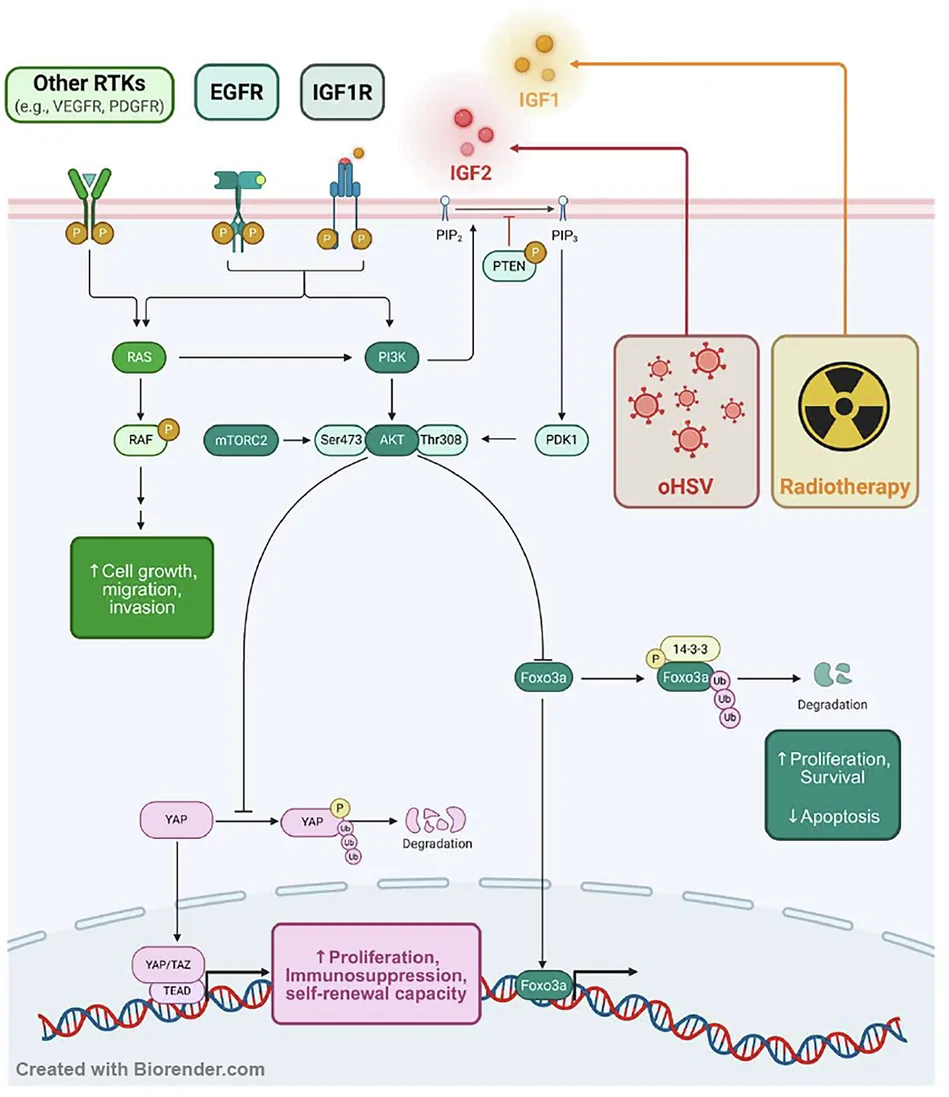

## Introduction

Oncolytic herpes simplex virus-1 (oHSV) is FDA-approved for melanoma [1, 2] and conditionally approved for glioblastoma (GBM); however, clinical responses remain limited [3]. Resistance arises from both tumor-intrinsic and -extrinsic mechanisms, including dysregulated interferon signaling, aberrant growth factor pathways, and pronounced tumor heterogeneity [4, 5]. Moreover, oHSV can paradoxically suppress anti-tumor immunity and activate pro-survival pathways such as PI3K/AKT and mTORC1, promoting tumor persistence [6, 7].

Our group recently discovered that oHSV infection stimulates insulin-like growth factor 2 (IGF2) secretion, which in turn activates the insulin-like growth factor 1 receptor (IGF1R)-AKT signaling throughout the tumor microenvironment (TME). This activation fosters immune suppression and diminishes the therapeutic efficacy of oHSV [8]. In addition, viral proteins such as Us3 and virus-induced host factors such as heparinase further augment these pro-survival signaling cascades [9, 10]. Together, these findings underscore a critical therapeutic challenge: how to preserve and enhance the immunogenic and oncolytic functions of oHSV while mitigating virus-induced, tumor-supportive signaling.

The insulin-like growth factor (IGF) system, a complex autocrine and paracrine network composed of ligands (IGF1, IGF2), receptors (IGFIR, IGF2R), and six IGF-binding proteins (IGFBP1 to IGFBP6), is frequently overexpressed in cancer and is associated with poor prognosis in GBM and breast cancer (BC) [11–15]. IGF1R signaling promotes metastasis and pre-metastatic niche formation [16, 17]. It is also implicated in driving resistance to radiation therapy (RTx), temozolomide (TMZ), and receptor tyrosine kinase (RTK) inhibitors in GBM and BC [11, 18–21]. Specifically, IGF1R activation following RTx and TMZ, standard of care (SOC) for intracranial malignancy, induces autophagy, stemness, senescence, and DNA repair, thereby attenuating treatment-induced apoptosis [22, 23]. Repeated RTx upregulates IGF1R expression and IGF1 secretion, further enhancing PI3K/Akt signaling and N-cadherin expression, which promotes tumor stemness and regrowth [19, 24]. Despite extensive interest in therapeutically targeting IGF1R signaling in GBM and BC, clinical inhibitors have shown limited success, likely due to compensatory activation of redundant RTKs such as EGFRwt, EGFRvIII, PDGFR, FGFR, and HER2 [11, 13, 25].

Herein, we expand upon our prior work demonstrating that oHSV-induced IGF2/IGF1R signaling promotes immunosuppression to explore its impact on cancer cell survival and resistance. We found that enhanced IGF1R activation occurs exclusively near sites of active viral replication, promoting tumor proliferation. While combining oHSV with IGF1R inhibition modestly enhanced therapeutic efficacy, the required doses impeded viral replication. Similarly, dual oHSV and RTx therapy showed limited benefit, further increasing IGF1R activation and inducing dose-dependent expression of the oncogenic and immunosuppressive factor YAP1 in resistant cells. Importantly, this IGF1R activation sensitized GBM and BC tumors to IGF1R-targeted therapy. Triple combination therapy with oHSV, RTx, and IGF1R blockade synergistically improved survival, abolished YAP1 expression, and reduced cancer cell self-renewal. Together, these findings identify IGF1R signaling as a key driver of resistance to oHSV therapy and support triple-combination strategies as a promising approach for the treatment of GBM and BC.

## Materials and methods

### Ethics statement

All animal studies were reviewed and approved by the Animal Welfare Committee (AWC), the Institutional Animal Care and Use Committee (IACUC) of the University of Texas Health Science Center at Houston (UTHealth), under protocol number AWC-24-0069. All procedures were conducted in accordance with institutional guidelines and applicable federal regulations for the care and use of laboratory animals.

### Cell lines and oHSV-1

All cell lines, primary GBM cells, and viruses used in this study are described in the Supplementary Materials and Methods.

### Cell proliferation assay, western blotting, immunohistochemistry, immunofluorescence, flow cytometry, soft agar colony forming assay, and qRT-PCR

All antibodies and primers (Supplementary Tables S1 and S2) are provided in the supporting data. Original full-length images of western blotting analysis and quantitative densitometric analyses are provided (Supplemental WB Quantification and Proofs). Detailed descriptions of these methods can be found in the Supplementary Materials and Methods.

### Animal studies

Six- to eight-week-old NSG, athymic nu/nu, and C57BL/6 mice were obtained from Jackson Laboratory (Bar Harbor, ME). All details of orthotopic stereotactic intracranial tumor, subcutaneous tumor, and orthotopic mammary fatpad implantation and treatment are described in the Supplementary Materials and Methods.

### Statistical analysis

Statistical analyses were performed using GraphPad Prism version 10 (GraphPad, San Diego, CA, USA RRID:SCR_002798)) or Python (Version 3.13). Unpaired student’s t-test was used to test continuous data with false discovery test described in the Supplementary Materials and Methods. To analyze survival data, Kaplan–Meier curves were compared using the log-rank test and the post hoc pairwise groups test was performed by Benjamini and Hochberg correction. Dose-response curves, synergy and sensitivity [26] calculations were made using SynergyFinder+ and summarized in Supplementary Table S3 [27]. All statistics are summarized in the Supplemental Statistics and the data files for upload to SynergyFinder+ are available in the Supplemental Synergy Data.

## Results

### oHSV infection promotes tumor proliferation through MAPK and PI3K-AKT signaling in GBM and breast cancer (BC)

**Fig. 1:**
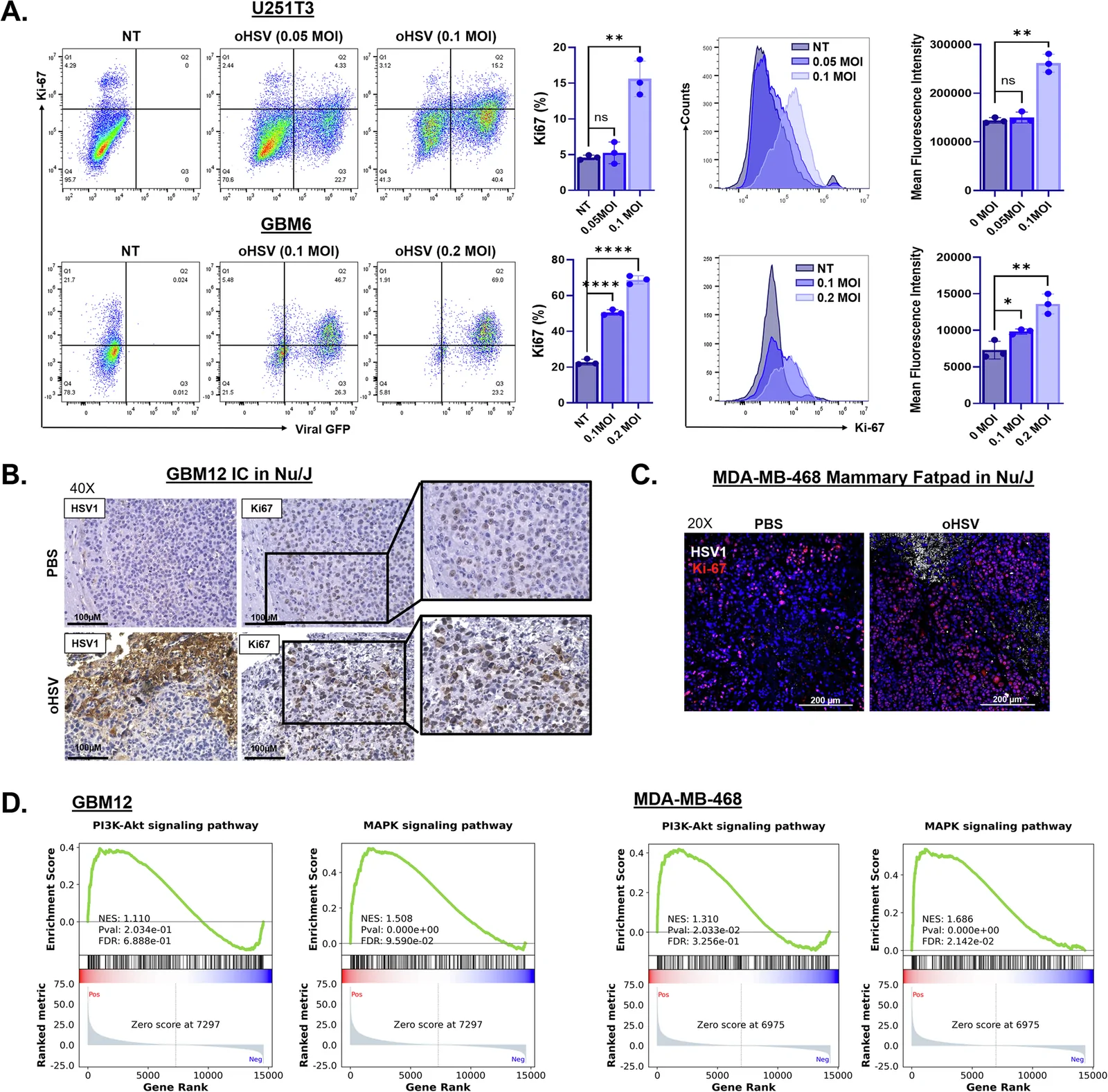
oHSV infection promotes proliferation via activation of MAPK and PI3K-AKT signaling in GBM and BC. **A** Flow cytometry analysis of Ki-67 in U251T3 glioma cell line (MOI = 0.05 and 0.1) and patient-derived primary GBM6 cells (MOI = 0.1 and 0.2) treated with or without oHSV for 48 and 24 hours, respectively (*n* = 3). Representative dot plots and quantification of Ki-67-positive cells are shown (left). Representative histograms of Ki-67 mean fluorescence intensity (MFI) with corresponding quantification are shown (right). Data are presented as mean ± SEM from triplicate experiments. **B** Representative histological analysis of oHSV-induced proliferation measured by Ki-67 staining in intracranial GBM12 tumor-bearing brain section from mice treated with or without oHSV (Magnification, ×40). **C** Representative fluorescent microscopy images stained for HSV1 (white), Ki-67 (red), and counterstained with DAPI (blue) (Magnification, ×20). **D** Gene set enrichment (GSEA) plots of PI3K-AKT and MAPK signaling in GBM12 and MDA-MB-468 BC cells (*n* = 4/group) treated with or without oHSV (MOI 0.1) for 16 hours, analyzed using previously published mRNA-sequencing data [8]. All data are presented as means ± SEM with *p < 0.01. **p < 0.005. ***p < 0.0005. ns not significant. All unspecified interactions were not significant.

We previously showed that IGF2 secreted from oHSV-infected tumor cells reduces therapeutic efficacy and that targeting IGF2 can reprogram the TME to enhance viro-immunotherapy. Here, we investigated whether oHSV-induced IGF1R signaling contributes to therapeutic resistance and whether its inhibition could improve oHSV efficacy. We used rHSVQ, an oHSV with γ34.5 deletion and ICP6 disruption, which shares a backbone with clinical candidates CAN-3110 (NCT03152318) and G207 (NCT00028158). Flow cytometry analysis demonstrates that the oHSV treatment enhanced tumor cell proliferation, as evidenced by increased Ki67 expression in subsets of both surviving infected and uninfected cells (Figs. 1A and S1). In vivo, oHSV treatment increased Ki67 staining in GBM12 and orthotopic MDA-MB-468 BC tumors, with Ki67-positive cells exclusively localized near regions of viral replication (Figs. 1B, C and S2–3). Gene set enrichment analysis (GSEA) of our previously published RNA sequencing (RNA-Seq) data revealed significant activation of MAPK, PI3K-Akt, and FoxO pathways in oHSV-infected GBM12 and MDA-MB-468 cells (Figs. 1D and S4), suggesting that while oHSV exploits these cascades for replication, they also drive tumor regrowth and immune evasion [8].

### oHSV infection induces IGF1R signaling in GBM and BC

**Fig. 2:**
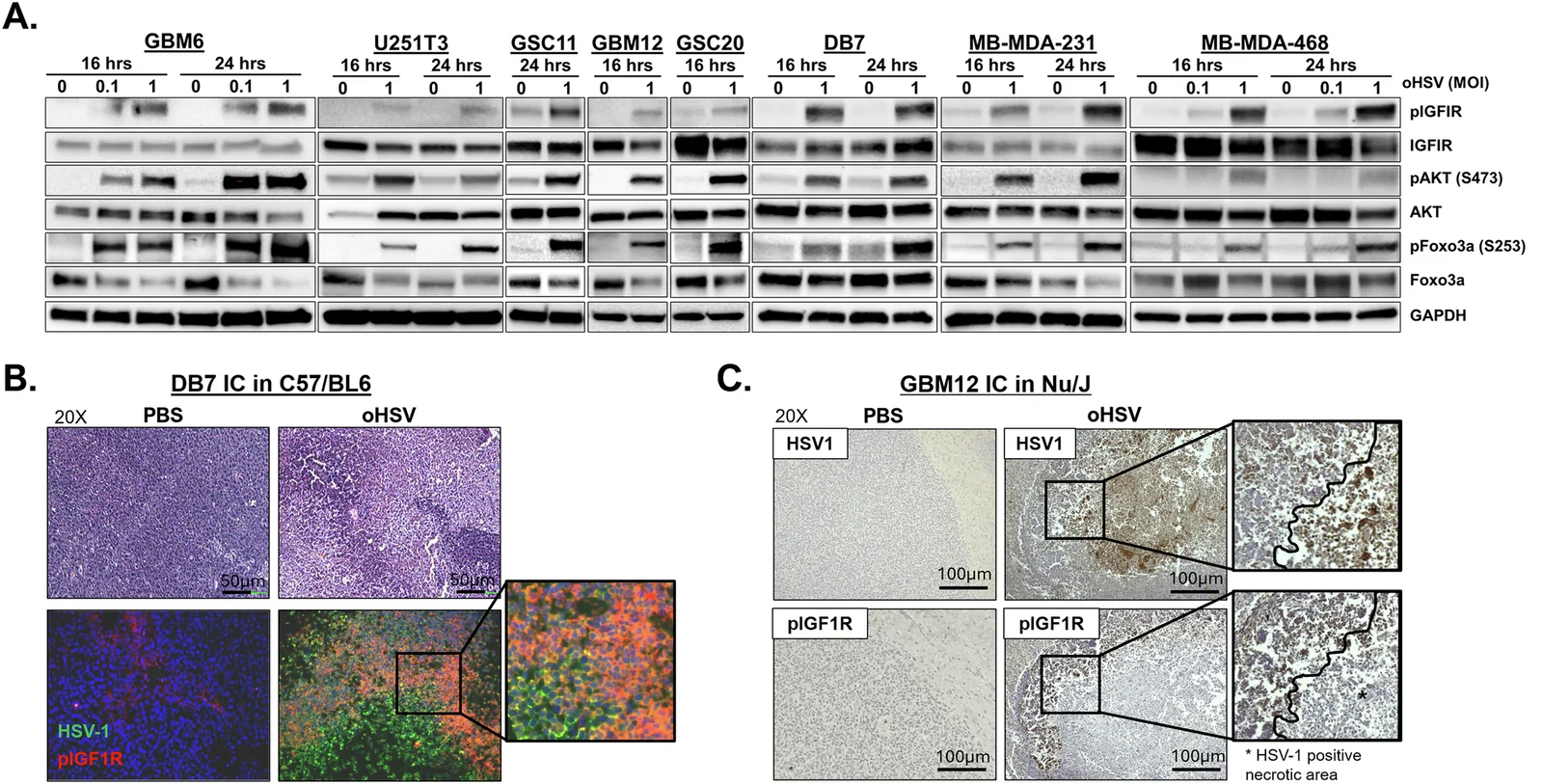
oHSV infection induces activation of IGF1R signaling in GBM and BC. **A** Western blot analysis of pathways downstream of IGF1R in patient-derived primary GBM cells and BC cell lines treated with or without oHSV (MOI = 0.1, 1) for 16 or 24 hours. Each experiment was performed with three independent biological replicates with the most representative samples shown. **B** H&E staining (top) and representative merged fluorescence microscopy images showing oHSV-induced activation of IGF1R signaling in DB7 BCBM tumor-bearing brain sections from mice treated intratumorally with or without oHSV (5 × 10^5^ pfu) (bottom). oHSV (green) and pIGF1R (red) were visualized, with nuclei counterstained with DAPI (blue). **C** Histological analysis of oHSV-induced IGF1R activation in intracranial GBM12 tumor-bearing brain sections treated with or without oHSV. (Magnification, ×20 for all microscopy).

While we previously demonstrated that IGF2 contributes to immune evasion [8], the impact of oHSV-induced IGF1R activation on tumor cell signaling remains unclear. Given that the MAPK, PI3K-Akt, and FoxO signaling cascades act downstream of IGF1R, we investigated their activation by western blot (Fig. 2A). Consistent with RNA-Seq data, oHSV treatment induced dose- and time-dependent increases in phosphorylation of IGF1R and Akt (S473) in BC, glioma cell lines, and patient-derived primary GBM cells. In addition, increased phosphorylation of FoxO3a (S253) supports our GSEA results of the canonical FoxO pathway activation, suggesting a role in promoting post-treatment tumor cell proliferation (Fig. 2A) [28]. Similarly, histology and immunofluorescence (IF) of DB7 murine breast cancer brain metastasis (BCBM) and orthotopic GBM12 brain tumors further confirmed enhanced IGF1R phosphorylation in and around regions of viral replication (Figs. 2B, C and S5 and 6). Notably, IGF1R activation persisted within both virus-infected and surrounding tumor areas despite extensive necrosis. Collectively, these findings suggest that oHSV-induced proliferation is largely driven by IGF1R signaling, potentially sensitizing tumors to IGF1R-targeted therapies.

### oHSV treatment sensitizes infected primary GBM and BC to IGF1R inhibition, enhancing anti-tumor efficacy

**Fig. 3:**
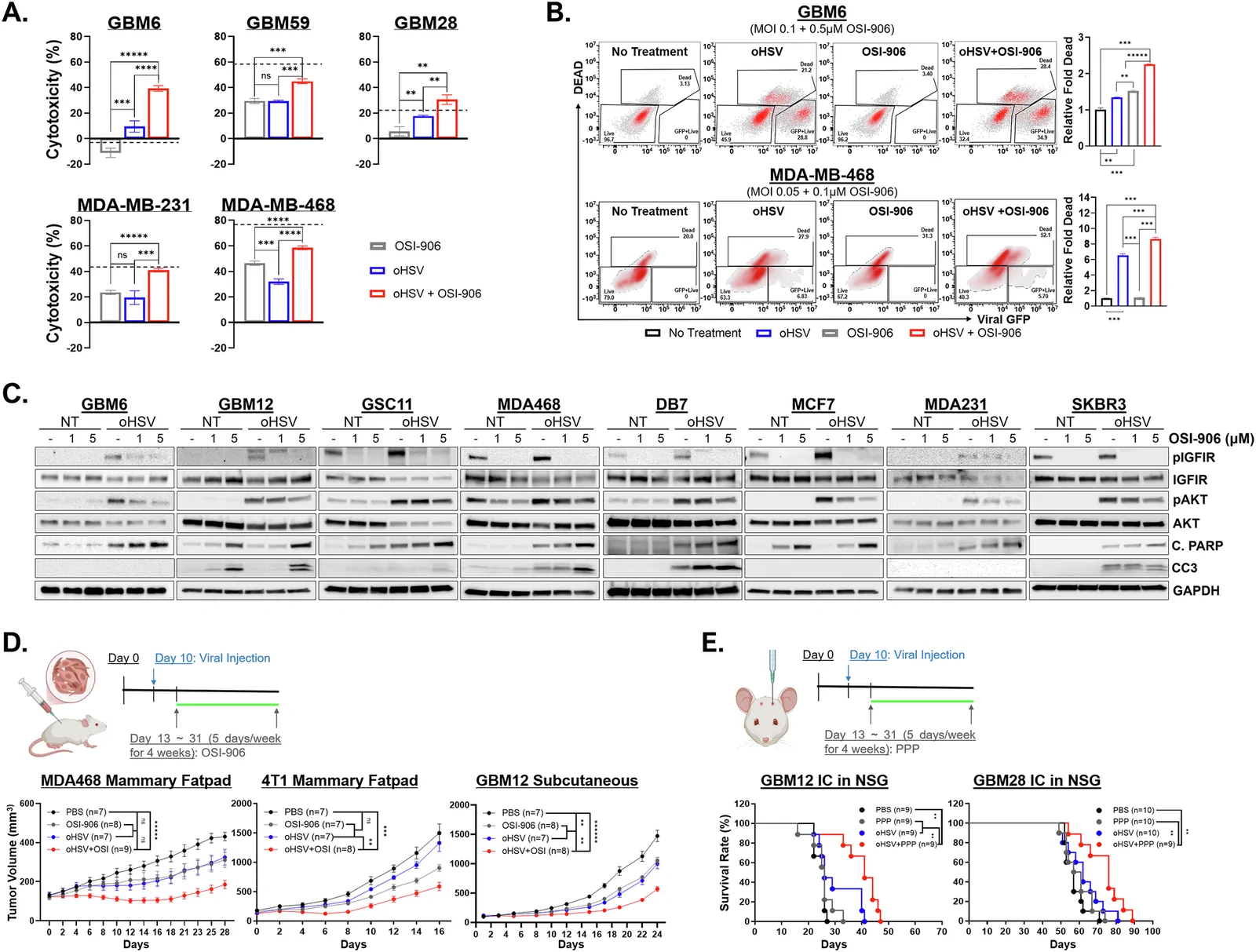
oHSV treatment sensitizes infected primary GBM and BC cells to IGF1R inhibition, enhancing anti-tumor efficacy. **A** MTT cell viability assay of patient-derived primary GBM and BC cells treated with or without oHSV, followed by treatment with DMSO solvent control or the small molecule IGF1R-inhibitor, OSI-906 (0.05, 0.1, 0.5, 1, or 5 μM) for 96 hours *(n* = 4/group). Cytotoxicity was determined relative to the untreated controls, and the dose combination demonstrating the greatest synergy is presented. Complete data can be found in the Supplemental “Triple Combination Synergy Table Input Files.” **B** Flow cytometry of Live/Dead staining and virally expressed GFP of GBM6 and MDA-MB-468 was performed 48 hours post-treatment with or without oHSV (MOI = 0.5 and 0.2, respectively) and with or without OSI-906 (0.1 or 0.5 μM OSI-906, respectively). The relative fold change in the percentage of dead cells was determined relative to the untreated/DMSO control group *(n* = 3/group). **C** Western blot analysis of patient-derived primary GBM12 and GSC11 cells treated with or without oHSV (MOI = 0.5 and 0.2, respectively) and BC cells treated with or without oHSV (MOI = 0.5) for 2 hours then either DMSO, 1, or 5 μM of OSI-906 for 18 hours. Each experiment was performed with three independent biological replicates with the most representative samples shown. **D** Patient-derived GBM12 cells were implanted subcutaneously, and BC cells (MDA-MB-468 and 4T1) were implanted into the mammary fat pad of athymic nude mice, which were then treated with PBS or oHSV intratumorally on days 10 and 13. Mice were then treated with 25 mg/kg OSI-906 5, days per week for 4 weeks, and tumor volume was monitored. Group sizes are indicated in the figures. **E** Kaplan–Meier survival curve of mice bearing intracranial GBM12 or GBM28 tumors treated intratumorally with PBS or oHSV 10 days after implantation, then treated with solvent control or 25 mg/kg PPP by oral gavage 5 days per week for 4 weeks. All data are presented as means ± SEM with *p < 0.01. **p < 0.005. ***p < 0.0005, ****p < 0.00005, ***** *p* < 0.000005. ns not significant.

To evaluate whether oHSV treatment sensitizes cancer cells to IGF1R inhibition, we used OSI-906, a potent orally available small-molecule IGF1R inhibitor. Cells were infected with oHSV for 1 hour and treated with OSI-906 (0.05–5 µM) or DMSO. A standard MTT assay at 96 hours showed that combination treatment modestly increased cancer cell killing compared to either monotherapy but was largely additive (dotted line) rather than synergistic (Fig. 3A). Live/dead cell staining for flow cytometry further confirmed an additive increase in MDA-MB-468 and GBM6 (Figs. 3B and S7). Western blot revealed effective inhibition of IGF1R signaling activation and dose-dependent increase in cleaved PARP and cleaved caspase 3 (CC3), indicating enhanced apoptotic cell death (Fig. 3C). However, OSI-906 also significantly impaired oHSV replication and propagation in a dose-dependent manner at OSI-906 concentrations ≥0.5 µM, as shown by viral GFP quantification using Cytation 5 Live Cell Imaging and plaque-forming assays (Figs. S8 and 9).

In vivo, dual therapy with oHSV and IGF1R blockade significantly reduced tumor burden in orthotopic MDA-MB-468 and 4T1 BC models, as well as in subcutaneously implanted GBM12 tumors, more effectively than monotherapy (Fig. 3D and Supplemental Statistics). In contrast, OSI-906 showed no benefit in intracranial GBM12 and GBM28 models due to poor blood–brain barrier (BBB) penetration (Fig. S10). Using picropodophyllin (PPP), a BBB-permeable IGF1R inhibitor, we observed significantly enhanced cancer cell killing compared to either monotherapy (Fig. S11). Combination therapy with oHSV and PPP also significantly prolonged survival, increasing median survival from 26 to 41 days in GBM12 and from 62 to 76 days in GBM28 (*P* = 0.0109 and 0.0347, respectively) (Fig. 3E and Supplemental Statistics). Collectively, these results suggest that IGF1R blockade enhances oHSV efficacy but remains insufficient for tumor eradication.

### Combined oHSV and RTx enhances IGF1R activation

**Fig. 4:**
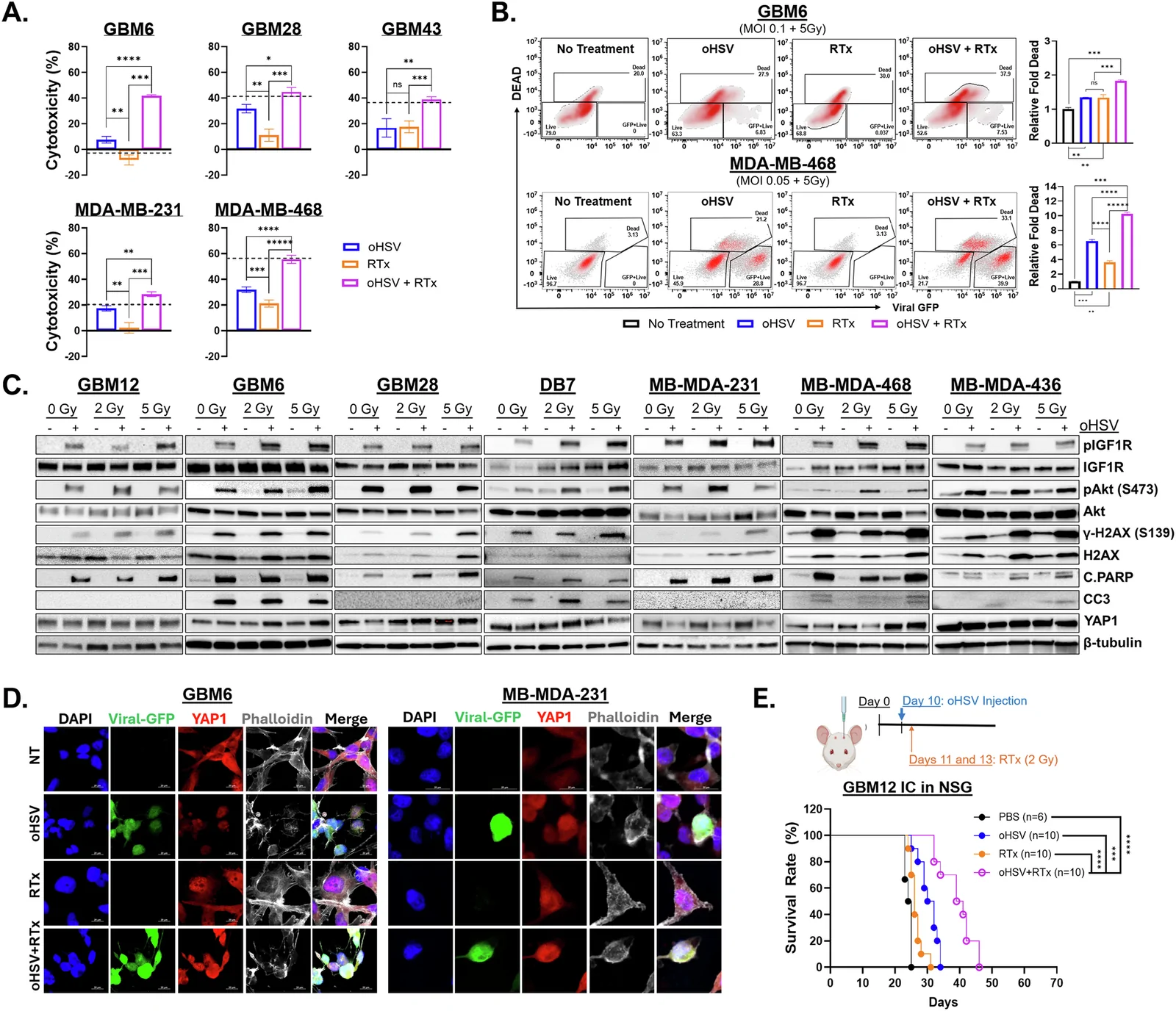
Combined oHSV and radiotherapy (RTx) enhances IGF1R activation. **A** MTT Cell Viability assay of patient-derived primary GBM and BC cells treated with or without oHSV and incubated for two hours then treated with or without RTx (2 or 5 Gy) for 96 hours *(n* = 4/group). Cytotoxicity was determined relative to the untreated samples and the dose combination with the greatest synergy presented. Complete data can be found in the Supplemental Triple Combination Synergy Table Input Files. **B** Flow cytometry of Live/Dead staining and virally expressed GFP of GBM6 and MDA-MB-468 was performed 48 hours post-treatment with or without oHSV (MOI = 0.5 and 0.2, respectively) and with or without RTx (5 Gy). The relative fold change in the percentage of dead cells was determined relative to the untreated/DMSO control group *(n* = 3/group). **C** Western blot analysis of patient-derived GBM cells and BC cells treated with or without oHSV (MOI = 0.2) then 1 hour later treated with or without radiotherapy (2, 5 Gy). Each experiment was performed with three independent biological replicates with the most representative samples shown. **D** Immunocytochemistry staining YAP1 (red), virally expressed GFP (green), and Phalloidin (white) of GBM6 and MDA-MB-231 performed 18 hours post-treatment with or without oHSV (MOI 0.2) and with or without RTx (5 Gy). **E** Kaplan–Meier survival curve of intracranial GBM12 tumor-bearing mice treated intratumorally with PBS or oHSV 10 days after implantation then treated with or without 2 Gy of RTx on days 11 and 13. All data are presented as means ± SEM with *p < 0.01. **p < 0.005. ***p < 0.0005, ****p < 0.00005. ns not significant. All unspecified interactions were not significant.

Radiotherapy (RTx) remains a cornerstone of the standard-of-care (SOC) for GBM and BC but induces IGF1/IGF1R signaling, triggering radio-resistance and recurrence [24]. As expected, combining oHSV with RTx significantly increased cytotoxicity across GBM and BC cells, either additively or synergistically, as measured by MTT assay (Fig. 4A and Supplemental Statistics). Live/dead cell staining confirmed additive enhancement of cell death (Figs. 4B and S7). Western blot revealed marked increases in γ-H2AX, cleaved PARP, and CC3, indicating enhanced DNA damage response and apoptotic cell death (Fig. 4C). Combination therapy further activated IGF1R-Akt signaling in an RTx dose-dependent manner (Fig. 4C).

RTx is known to induce YAP1 expression, a transcription factor that promotes radio-resistance, stemness, and immunosuppression [29–31]. Consistent with this, we observed RTx dose-dependent upregulation of YAP1 expression and IGF1R activation (Fig. 4C), along with increased nuclear localization, particularly in oHSV-resistant cells (Fig. 4D). Furthermore, GSEA revealed significant enrichment of Hippo pathway-associated gene signatures following oHSV infection in both GBM12 and MDA-MB-468 cells (Fig. S12), suggesting modulation of Hippo signaling. Consistent with this, we observed increased nuclear localization of YAP1 (Fig. 4D), indicating YAP1 pathway engagement in response to oHSV treatment. Notably, the combination treatment with oHSV and RTx further enhanced YAP1 nuclear localization in GBM6 and MDA-MB-231 (Fig. 4D). Kaplan–Meier analysis of GBM12 tumor-bearing mice showed improved survival with combination therapy (median survival: oHSV = 31 days; RTx = 26 days; combination = 40 days, *P* < 0.001 vs. oHSV, *P* < 0.0001 vs. RTx) (Fig. 4E). Collectively, these findings demonstrate that while oHSV+RTx enhances therapeutic efficacy, it simultaneously promotes IGF1R/YAP1 signaling, which may drive residual resistance.

### Combined RTx and oHSV treatment synergizes with IGF1R inhibition in vitro and in vivo

**Fig. 5:**
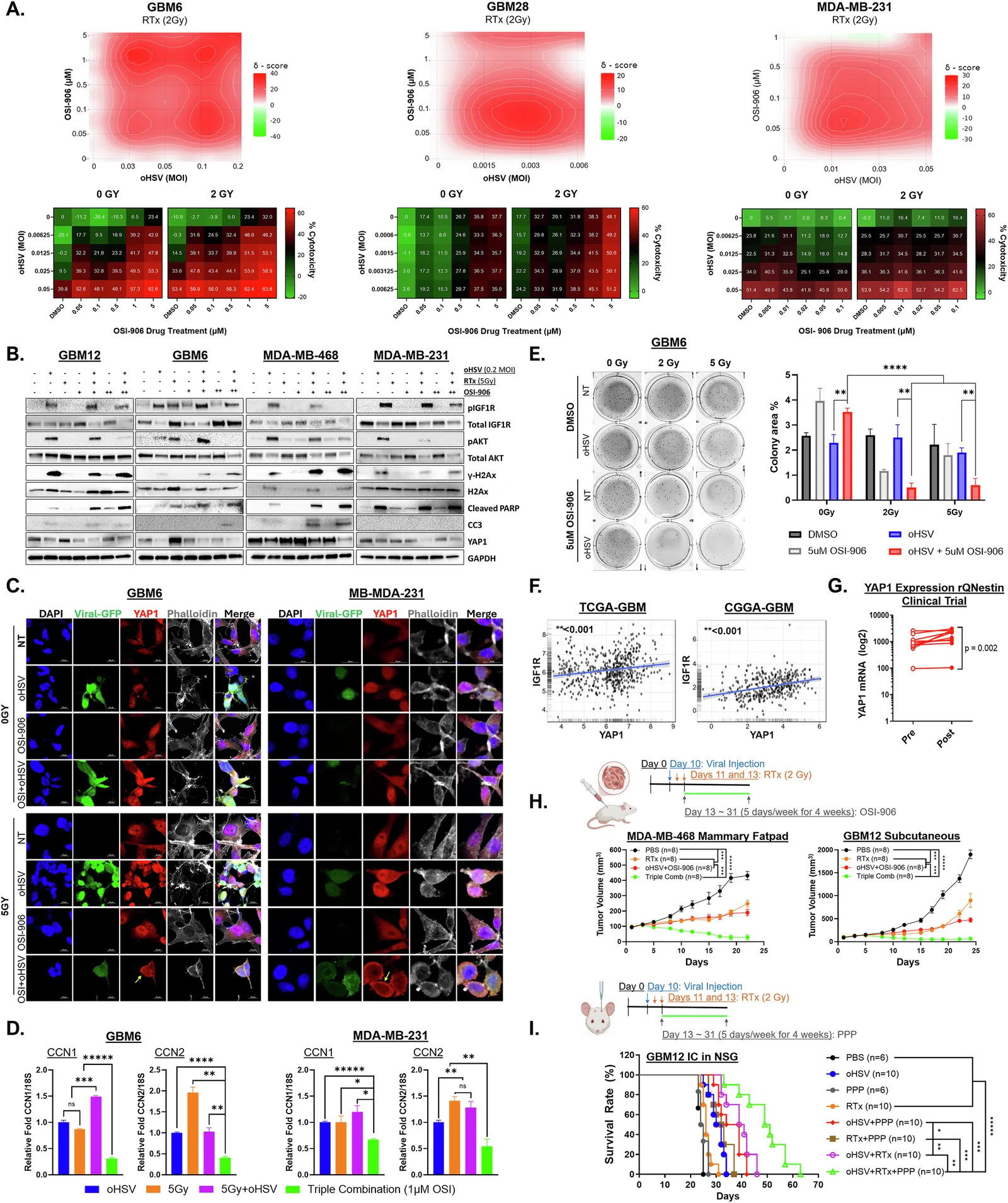
Combined RTx and oHSV treatment synergizes with IGF1R inhibition in vitro and in vivo. **A** MTT Cell Viability assay of patient-derived primary GBM and BC cells treated with or without oHSV, then with or without RTx (2, 5 Gy), then 1 hour later treated with DMSO solvent control or OSI-906 (0.05, 0.1, 0.5, 1, or 5 μM) for 96 hours *(n* = 4/group) and the dose combination with the greatest synergy is presented. Complete data can be found in the Supplemental Triple Combination Synergy Table Input Files. Bliss synergy interaction landscapes at 2 Gy are depicted (top) along with heatmaps of cytotoxicity relative to no treatment. **B** Western blot analysis of patient-derived primary GBM cells and BC cell lines treated with triple combination therapy or monotherapy as in (**A**). Each experiment was performed with three independent biological replicates, with the most representative samples shown. **C** Immunocytochemistry staining YAP1 (red), virally expressed GFP (green), and Phalloidin (white) in GBM6 and MDA-MB-231 performed 18 hours post-treatment with or without oHSV (MOI 0.2), with or without RTx (5 Gy), and with or without OSI-906 (5 µM). **D** RT-qPCR of MDA-MB-231 and GBM6 cells treated with or without oHSV (0.2 and 0.1 respectively), then with or without RTx (5 Gy), then with or without OSI-906 (1 µM) (*n* = 3/group). CCN1 and CCN2 mRNA expression was normalized to 18S ribosomal RNA and relative fold expression change from oHSV alone presented. **E** Representative wells of soft agar colony-forming assay of GBM6 cells treated as in (**A**) (left) and total area covered by colonies quantified as a percentage of total area using the ColonyArea plugin for FIJI [60]. **F** Correlation between log2 mRNA expression of IGF1R and YAP1 acquired using the TCGA-GBM (left) and CGGA-GBM (right) data sets. **G** YAP1 gene expression levels pre- and post-rQNestin34.5v2 treatment in 14 recurrent GBM patients (ClinicalTrials.gov, NCT03152318). **H** MDA-MB-468 cells were implanted into the mammary fatpad and patient-derived GBM12 cells were implanted subcutaneously in athymic nude mice then treated with PBS or oHSV intratumorally on day 10 and 13. Mice were then treated with or without RTx (2 Gy) followed by 25 mg/kg OSI-906 5 days per week for 4 weeks and tumor volume was measured (mm^3^). Group sizes are shown in the figure. **I** Kaplan–Meier survival curve of intracranial GBM12 tumor-bearing mice treated intratumorally with PBS or oHSV 10 days after implantation then with or without RTx (2 Gy), then treated with solvent control or 25 mg/kg PPP by oral gavage 5 days per week for 4 weeks. All data are presented as means ± SEM with *p < 0.01. **p < 0.005. ***p < 0.0005, ****p < 0.00005, *****p < 0.000005. ns not significant. All unspecified interactions were not significant.

We next investigated whether RTx- and oHSV-induced activation of IGF1R signaling sensitizes tumor cells to IGF1R-targeted therapy. Cells were infected with oHSV (0.001–0.2 MOI), treated one hour later with RTx (0 or 2 Gy), and then exposed to OSI-906 (0.05–5 µg/ml). Cytotoxicity measured by MTT assay at 96 hours revealed strong synergy with triple combination in patient-derived GBM cells (GBM6, GBM28) and BC cells (MDA-MB-231), as measured by SynergyFinder+ analysis (Figs. 5A and S13, Table S3) [32]. Similarly, the combined sensitivity score (CSS) and relevant inhibition (RI) score confirm that dual oHSV and RTx therapy sensitize tumors to IGF1R inhibition (Table S2). Notably, while Bliss synergy scores indicated overall antagonism in MDA-MB-231 (mean = −3.74, *P* = 3.56e-3) at higher OSI-906 doses, marked synergy was observed at lower dosage (<µM concentrations), particularly at lower oHSV and RTx doses. The Loewe model confirmed strong synergy (mean = 15.12, *P* = 1.77e-18), while dual therapy combinations showed antagonistic interactions not present with the triple combination (Fig. S13B).

Mechanistically, triple combination therapy reduced IGF1R-Akt activation, enhanced DNA-damage responses, and increased PARP and Caspase-3 cleavage, with a consistent dose-dependent decrease in YAP1 expression across all cell types tested (Fig. 5B). Notably, triple combination therapy markedly reduced YAP1 nuclear localization and reduced transcription of the YAP1 target genes CCN1 and CCN2 (Fig. 5C, D). Functional assays further demonstrated markedly reduced colony formation and proliferation (Figs. 5E and S14).

Analysis of the Rembrandt and TCGA-GBM databases supported these findings. YAP1 expression was elevated in GBM versus non-tumor brain tissue (Fig. S15A, B, left) and negatively correlated with patient survival, particularly in female patients (Fig. S15A, B, right). Notably, there was no significant correlation with survival in male patients in the TCGA-GBM, making the combined data insignificant (Fig. S15C). Furthermore, YAP1 positively correlated with IGF1R gene expression in GBM in both the TCGA and Chinese Glioma Genome Atlas (CGGA) datasets (Fig. 5F). Importantly, tumor samples from 12 recurrent GBM patients treated with rQNestin34.5 v.2 (ClinicalTrials.gov: NCT03152318) showed increased YAP1 expression in 7 patients (58.33%; *P* = 0.0020), underscoring the clinical relevance of YAP1 upregulation in response to oHSV therapy (Fig. 5G) [33].

Finally, the triple combination therapy markedly reduced tumor burden in orthotopic MDA-MB-468 xenografts, with mean tumor volumes by day 22 of 432.21 ± 27.3 mm³ in controls, 248.4 ± 23.3 mm³ with RTx (42.5% inhibition, *P* < 0.001), 183.8 ± 20.7 mm³ with oHSV+OSI-906 (57.4% inhibition, *P* < 0.001), and 49.6 ± 23.3 mm³ with the triple combination therapy (88.5% inhibition, *P* < 0.000001), and complete tumor regression in 5 out of 8 mice (Fig. 5H left). Similar results were seen in subcutaneous GBM12 models, including complete tumor regression in 2 out of 8 mice (*P* < 0.001 vs RTx or oHSV+OSI-906) (Fig. 5H right). Kaplan–Meier survival analysis further demonstrated that the triple combination therapy significantly extended survival in intracranial GBM12-bearing mice (median = 50 days) compared to dual therapies (*P* < 0.0001 vs. dual therapies; median survival: oHSV+PPP = 36.5 days; oHSV+RTx = 40 days; or RTx+PPP = 32.5 days) (Fig. 5I and Supplemental Statistics). Collectively, these findings demonstrate that combining RTx, oHSV, and IGF1R inhibition synergistically enhances antitumor efficacy in GBM and BC, reduces the effective dose of each agent, and overcomes IGF1R-mediated resistance.

## Discussion

Insulin-like growth factor 1 receptor (IGF1R) signaling has emerged as a critical driver of therapeutic resistance to receptor tyrosine kinase (RTK) inhibitors, chemotherapy, and radiotherapy (RTx) in glioblastoma (GBM) and breast cancer (BC), fueling sustained interest in IGF1R-targeted therapies [12, 19, 24, 34]. The clinical feasibility of this approach is underscored by the FDA approval of the IGF1R inhibitor teprotumumab (TEPEZZA^®^) in 2020 for thyroid eye disease [35–37]. However, despite extensive preclinical and clinical investigation, no IGF1R inhibitors have been approved for cancer treatment, likely due to pathway redundancy and compensatory kinase activation [18]. Our findings suggest that IGF1R remains an exploitable vulnerability when integrated into rationally designed multimodal therapies. Specifically, combining oHSV and RTx, two modalities that converge on IGF1R-mediated resistance, may enhance therapeutic efficacy and overcome tumor-intrinsic resistance.

Currently, more than 200 clinical trials are evaluating oncolytic viruses (OVs) as monotherapies or in combination regimens, leading to the approval of oHSV talimogene laherparepvec (T-VEC; IMLYGIC®, Amgen Inc) for metastatic melanoma [2, 38, 39]. Despite this progress, the efficacy of oHSV remains limited by both tumor-intrinsic and extrinsic resistance. One major tumor-intrinsic barrier is the compensatory activation of pro-survival pathways downstream of RTKs, such as PI3K/AKT, mTORC1, and MAPK signaling, that promote tumor regrowth. Meticulous investigation of these resistance mechanisms is crucial to optimize next-generation viral constructs and rationally designed combination regimens [4, 5, 40].

We previously demonstrated that oHSV infection induces IGF2 secretion, activating IGF1R signaling and reshaping the tumor immune microenvironment (TIME) by recruiting immunosuppressive myeloid cells (e.g. mMDSCs, gMDSCs) [8]. Herein, we extend these findings by showing that oHSV triggers IGF2/IGF1R activation not only in infected tumor cells but also in neighboring uninfected cells, promoting tumor regrowth and therapeutic resistance. This pro-survival response mirrors the effect of RTx, which similarly upregulates IGF1/IGF1R signaling [19, 24]. Combining oHSV with RTx further amplified IGF1R activation, creating an exploitable adaptive therapeutic vulnerability that sensitizes tumors to IGF1R inhibition. Indeed, IGF1R blockade suppressed IGF1R signaling and YAP1 activity, suppressing tumor cell proliferation, self-renewal, and enhancing survival in orthotopic GBM and BC models (Figs. 5B–E and 5H, I).

While the observed synergy would reduce the required dose of each agent, systemic IGF1R inhibition has been associated with dose-limiting toxicities in clinical trials, raising concerns regarding tolerability. Furthermore, treatment sequencing is a key determinant of both efficacy and safety in combination regimens. We employed a rationale-driven sequential regimen in which oHSV was administered first, followed by RTx and IGF1R blockade. This design exploits the dynamic emergence of IGF1R signaling while limiting concurrent exposure to targeted inhibition. While this approach demonstrated synergistic efficacy, further studies are needed to optimize treatment sequencing and define the balance between therapeutic efficacy and toxicity in clinical settings.

YAP and TAZ, key transcriptional co-activators and effectors of Hippo signaling, are intimately associated with tumorigenesis, proliferation, stemness, recurrence, immunosuppression, and RTx resistance in cancer broadly [31, 41–44]. Although IGF1R-mediated regulation of YAP1 has only recently been implicated in GBM [45], the underlying mechanisms remain incompletely defined. In triple-negative breast cancer (TNBC), IGF1/IGF1R/FAK signaling decreases YAP1 phosphorylation, promoting nuclear localization and driving aggressive phenotypes [46, 47]. Similarly, in diffuse large B-cell lymphoma, IGF1R signaling represses MLK1/2 activity to prevent YAP1 phosphorylation [48]. Importantly, YAP1 enhances IGF1 and IGF1R expression in both GBM and BC brain metastases (BCBM), creating a feedforward loop driving therapeutic resistance and progression [46]. Similarly, in medulloblastoma, RTx-induced YAP1 activity has been shown to induce IGF2 secretion. Moreover, knockdown of IGF2 abolished YAP-induced proliferation and pro-survival signaling [49]. This effect was particularly evident in GBM cells harboring the EGFRvIII mutation (Fig. 5D), which exhibit elevated YAP/TAZ activity and heightened sensitivity to YAP/TAZ-targeted therapies [50]. Together, these findings support a functional IGF1R-YAP1 signaling axis in tumor progression and therapy resistance. Further studies are warranted to define the precise mechanism of IGF1R-mediated YAP1 activation in viro-immunotherapy and to determine the therapeutic impact of direct YAP/TAZ inhibition in GBM.

Importantly, the IGF1R-YAP1 signaling axis may reinforce immunosuppression within the TIME, further limiting viro-immunotherapy efficacy. IGF1R and YAP1 signaling promote the recruitment of TAMs, which secrete pro-tumorigenic cytokines and reinforce IGF1R/YAP1 activation [51, 52]. M2-like TAMs and microglia, for example, secrete IGF1, amplifying this feedforward loop [8, 11, 25, 53–55]. In the adaptive immune context, YAP1 promotes the differentiation of regulatory T cells (Tregs) [56] while suppressing CD8+ cytotoxic T cell infiltration and inducing PD-L1 expression, thereby reducing responses to immune checkpoint blockade (ICB) [52, 57, 58]. Thus, IGF1R/YAP1 signaling may represent a key mechanism of immune evasion and attractive therapeutic target to enhance viro-immunotherapy.

Recent studies further highlight the importance of neuron–tumor interactions in GBM and brain metastases, where tumor cells form pseudo-synaptic connections and exploit neuron-derived monoamine signaling to enhance growth and resistance. Neuroactive pathways converge on AKT/mTORC1, AMPK, YAP signaling. For example, moxidectin has been shown to inhibit MEK/ERK/YAP signaling, enhance antigen presentation in tumor-draining lymph nodes, and reduce TAM infiltration in GBM [59]. In this context, our findings suggest a potential convergence between neuron-derived signaling and IGF1R-driven YAP1 activation. Although not directly examined in the current study, integrating neuroactive signaling modulation with the current triple combination therapy may enhance therapeutic efficacy and warrants further investigation.

In conclusion, this study uncovers IGF1R-driven YAP1 activity as a critical adaptation following oHSV and RTx, positioning it as a promising therapeutic target. Disrupting this pathway may enhance tumor control and alleviate immunosuppression. Future studies should delineate how IGF1R/YAP1 modulation reshapes the TIME and determine whether its inhibition can overcome immune evasion to maximize the therapeutic benefit of virotherapy.

## Supplementary information

Supplement

Supplementary Tables

Supplemental Table S1

Supplementary Statistics

Triple Combination Synergy Input- GBM28

Triple Combination Synergy Input- MDA-MB-231

## Data Availability

The data generated in this study are available within the article and its supplementary data files.
