# Supplementary material for: Overcoming IGF1R-mediated resistance to oncolytic HSV1 and radiotherapy via triple combination therapy
Source: Cell Death Dis. 2026 Jul 17;17(1):881. doi: 10.1038/s41419-026-09090-5 (PMC13658246; doi:10.1038/s41419-026-09090-5)

Overcoming IGF1R-Mediated Resistance to Oncolytic HSV1 and Radiotherapy via Triple Combination Therapy

Alexandra A. Miller[1,2*], Min-Hye Noh[1*], Jin Muk Kang[1,3], Jiyeon Kim[1], Lily Nguyen[1,2], Amanda S. Kouaho[1], Grace Nguyen[1,4], Minxin Huang[1,5], Stephanie M. Bean[1], Joshua Davis[1], Matthew P. Mullarkey[1], Sunil Krishnan[1,2], Zhongming Zhao[2,6], E. Antonio Chiocca[7], Tae Jin Lee[1,2#], and Ji Young Yoo[1,2#]

[1]Department of Neurosurgery, McGovern Medical School, The University of Texas Health Science Center at Houston, Houston, TX, 77030, USA. [2]The University of Texas MD Anderson Cancer Center UTHealth Houston Graduate School of Biomedical Sciences, Houston, TX 77225, USA. [3]Department of Pediatric Hematology & Oncology, University Hospitals Cleveland Medical Center, Cleveland, OH, 44106, USA. [4]Department of Pathology, University of Texas Southwestern Medical Center, Dallas, TX 75390, USA. [5]Molecular and Cellular Pharmacology Program, Department of Pharmacological Sciences, Renaissance School of Medicine at Stony Brook University, Stony Brook, NY 11794, USA [6]Center for Precision Health, McWilliams School of Biomedical Informatics, The University of Texas Health Science Center at Houston, Houston, TX 77030, USA. [7]Harvey W. Cushing Neuro-oncology Laboratories, Department of Neurosurgery, Brigham and Women's Hospital and Harvard Medical School, Boston, MA USA.

[*]These authors contributed equally.

Western Blot Quantification

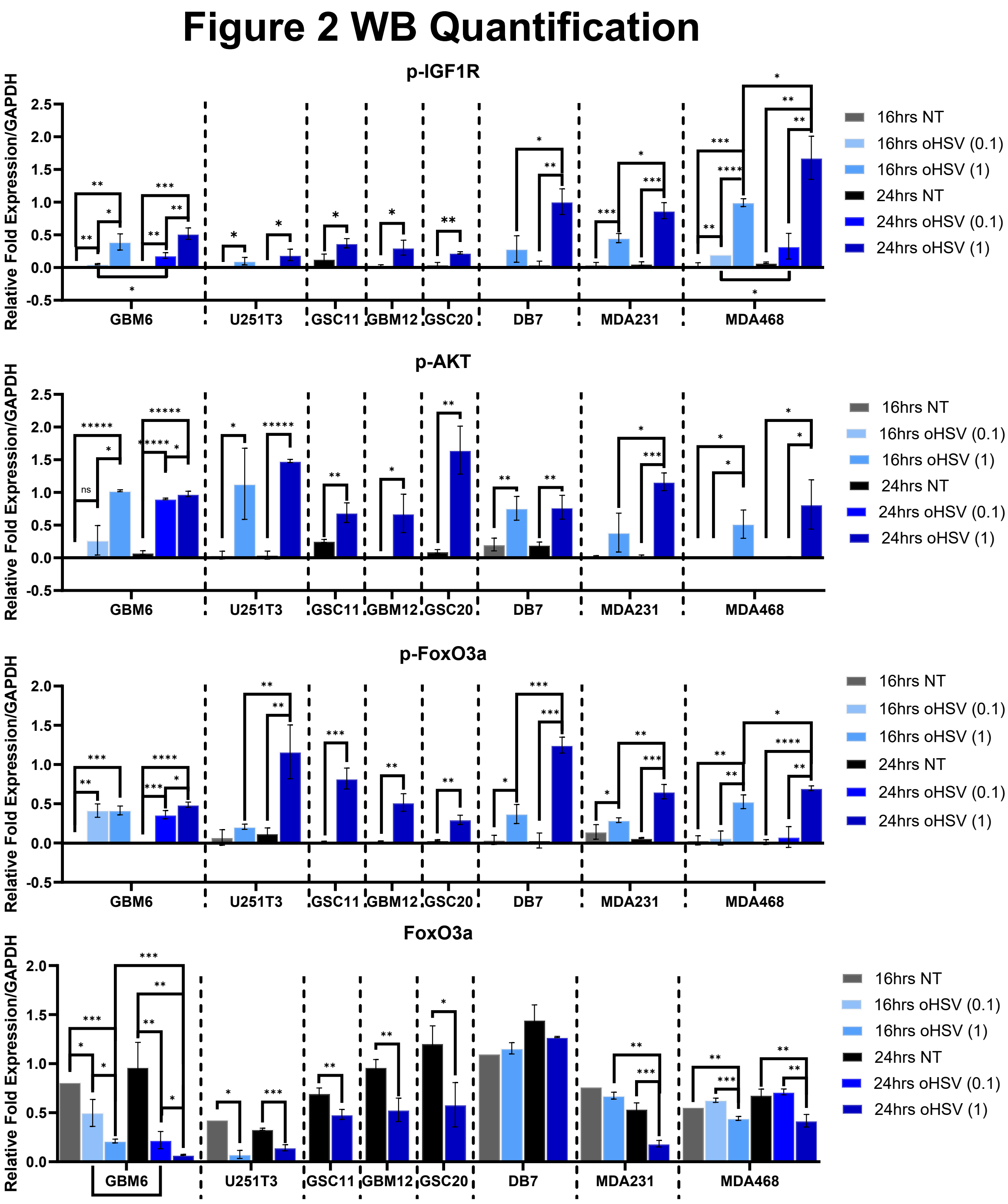

Figure 3 WB Quantification

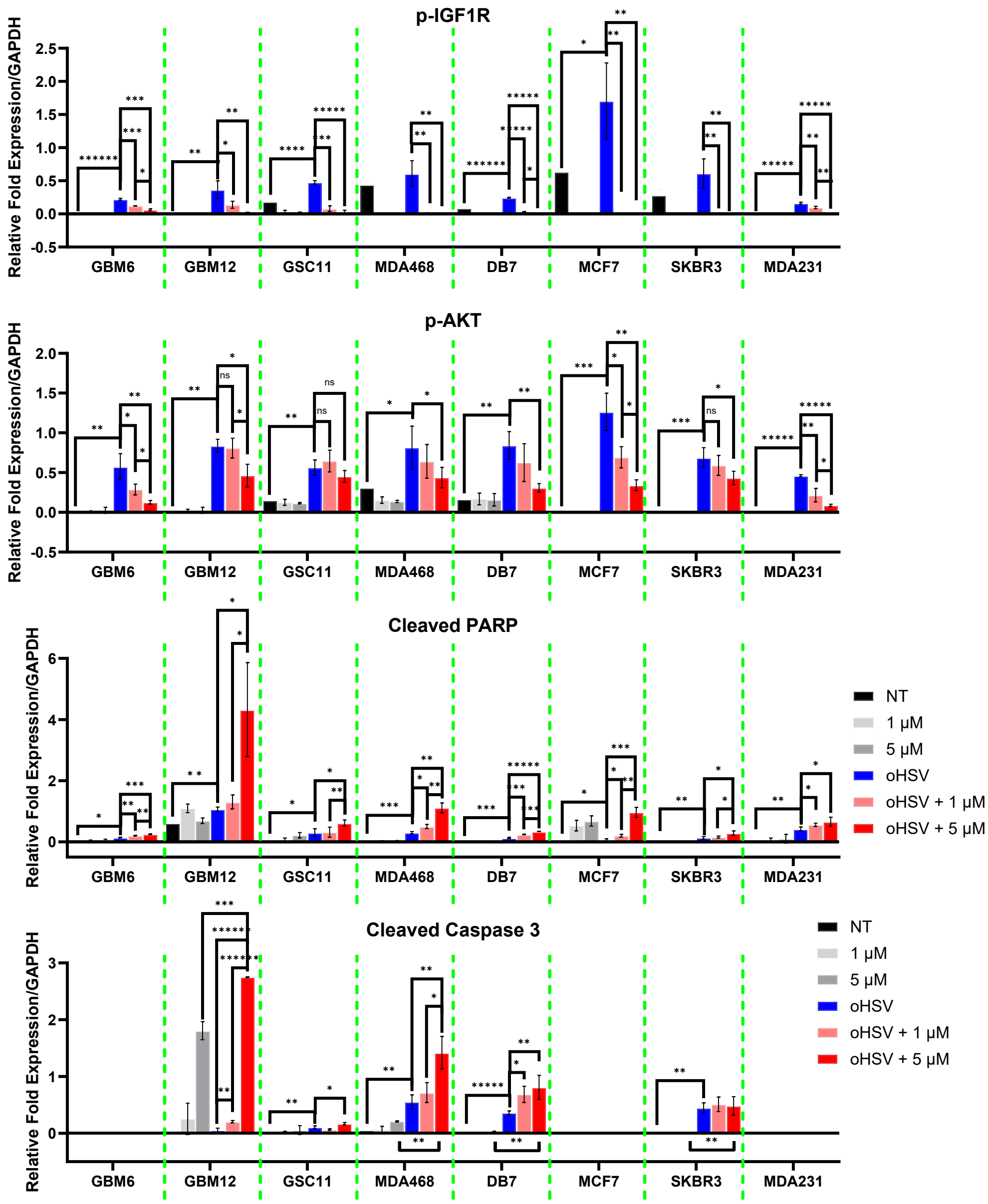

Figure 4 WB Quantification

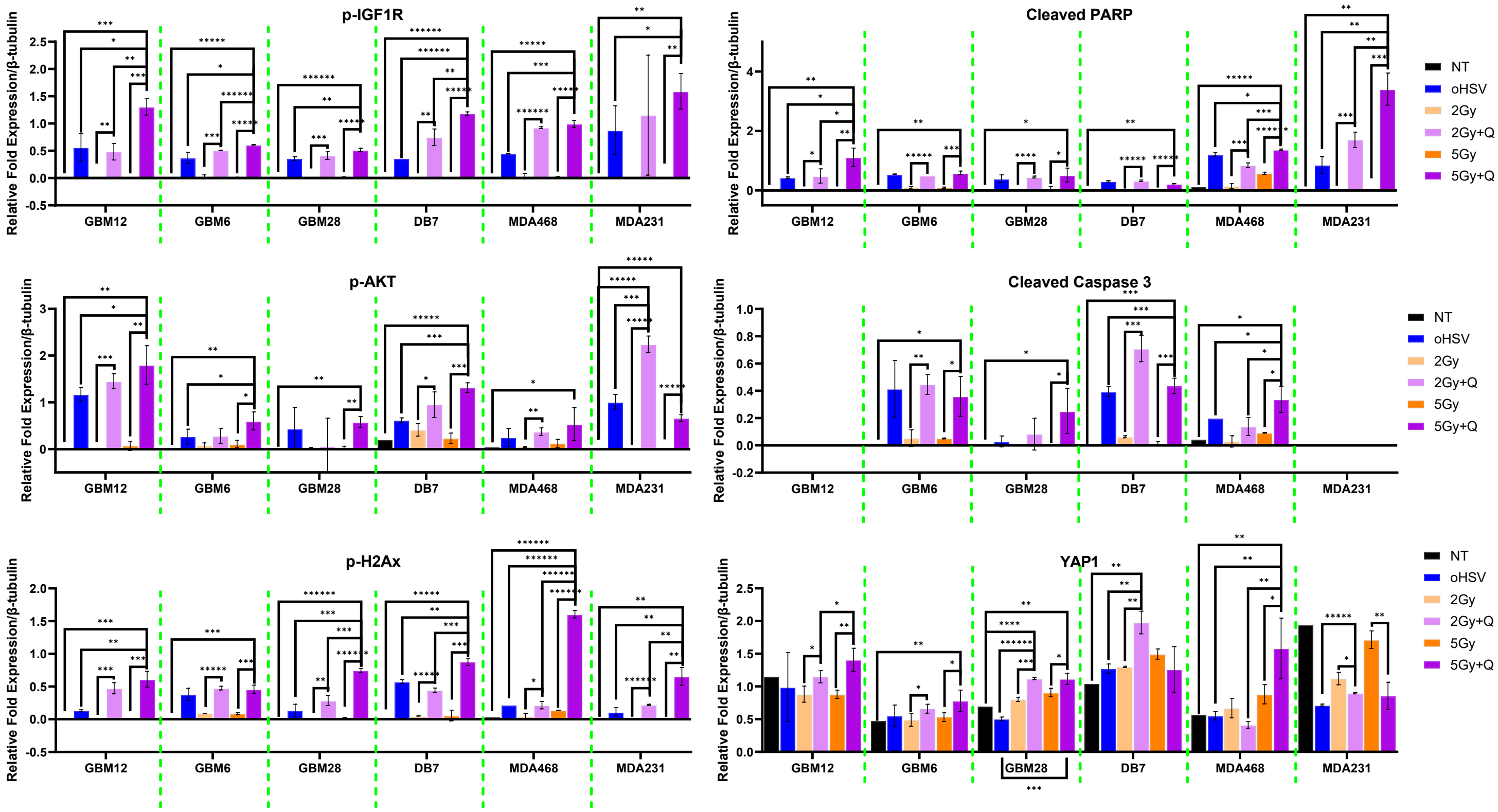

Figure 5 WB Quantification

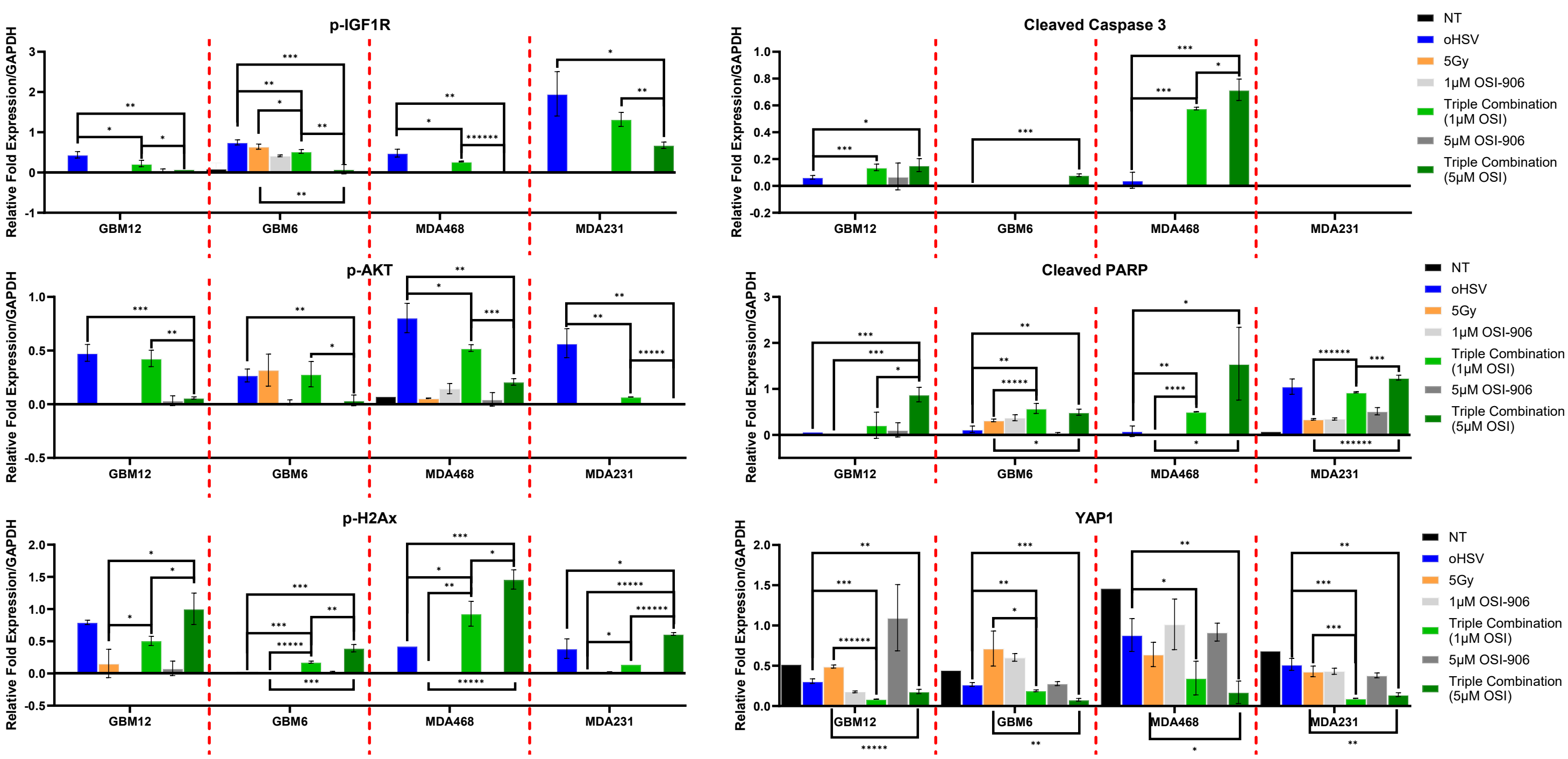

Supplementary Western Blot Proofs.

Original image of Western blotting analysis. Sample orders are given from left to right.

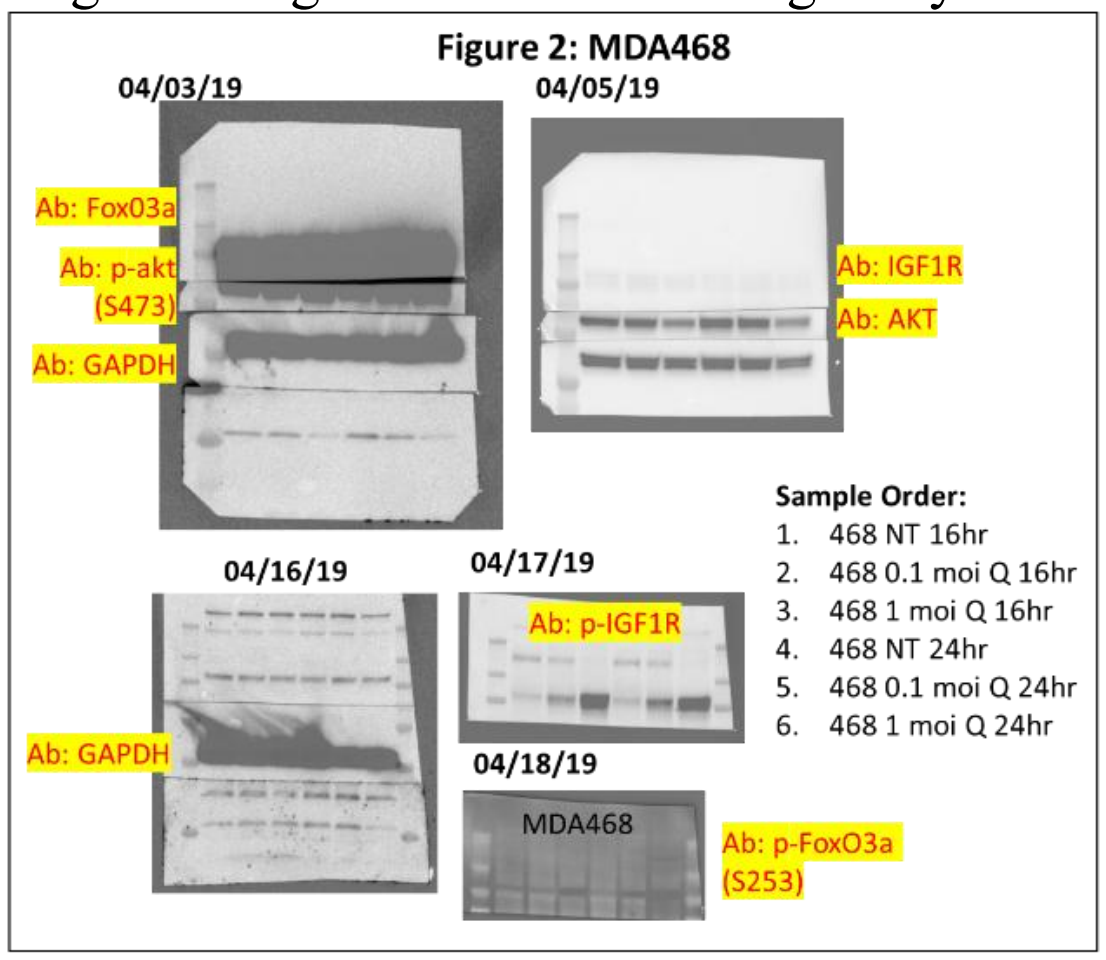

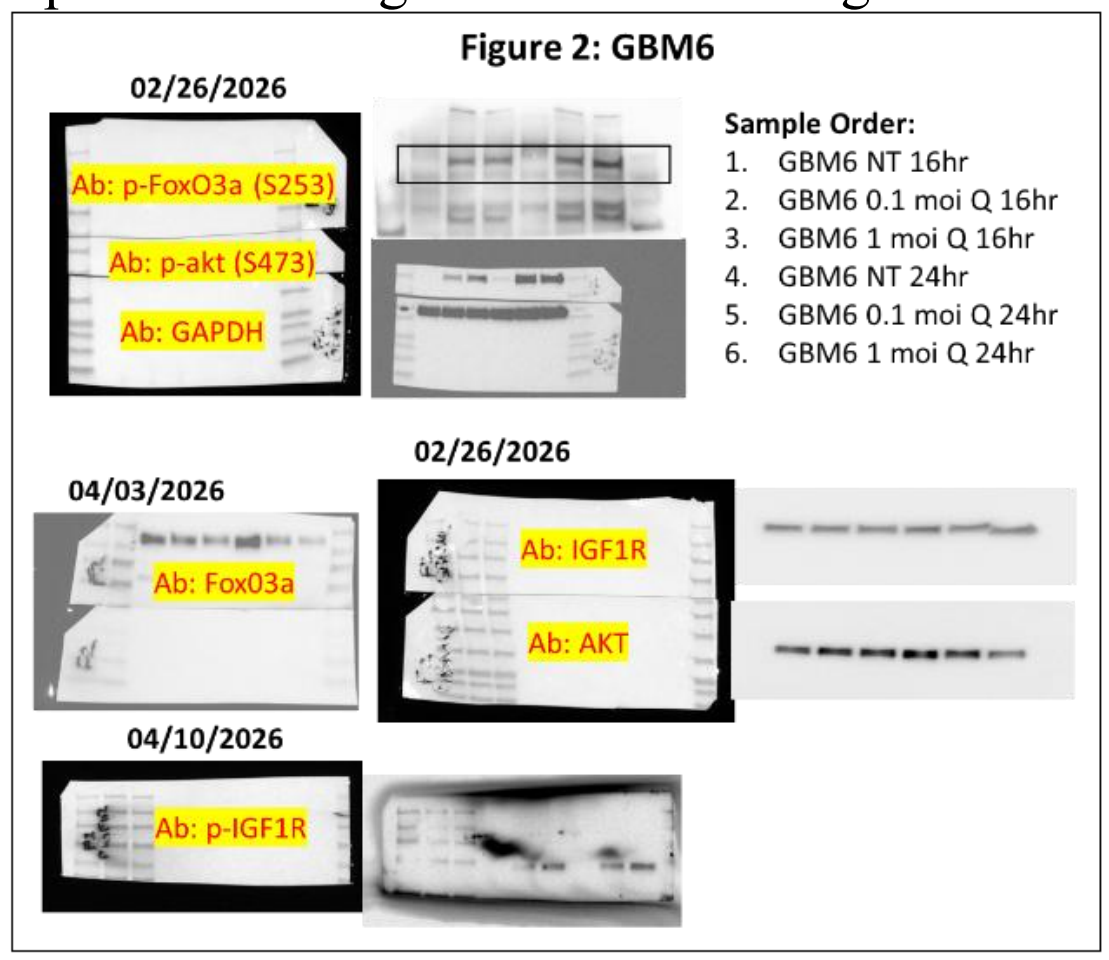

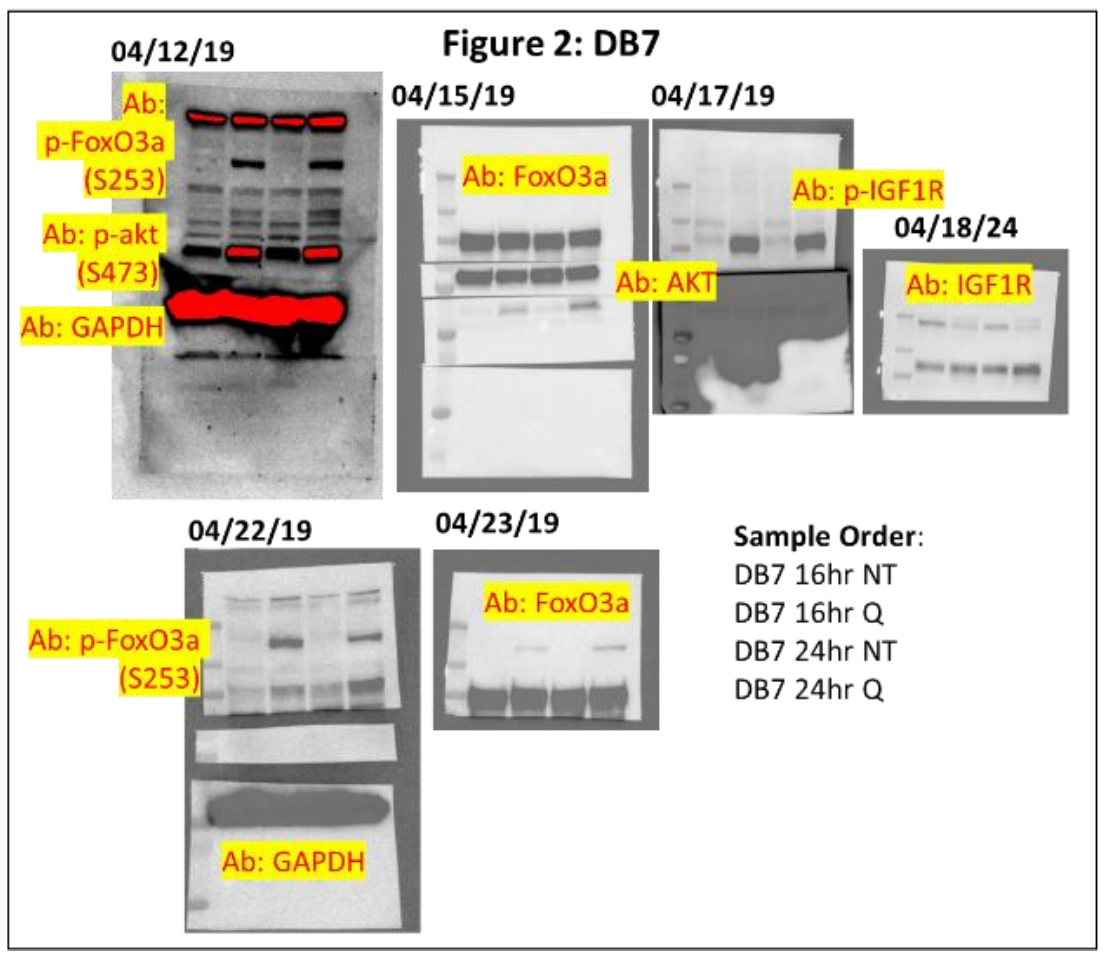

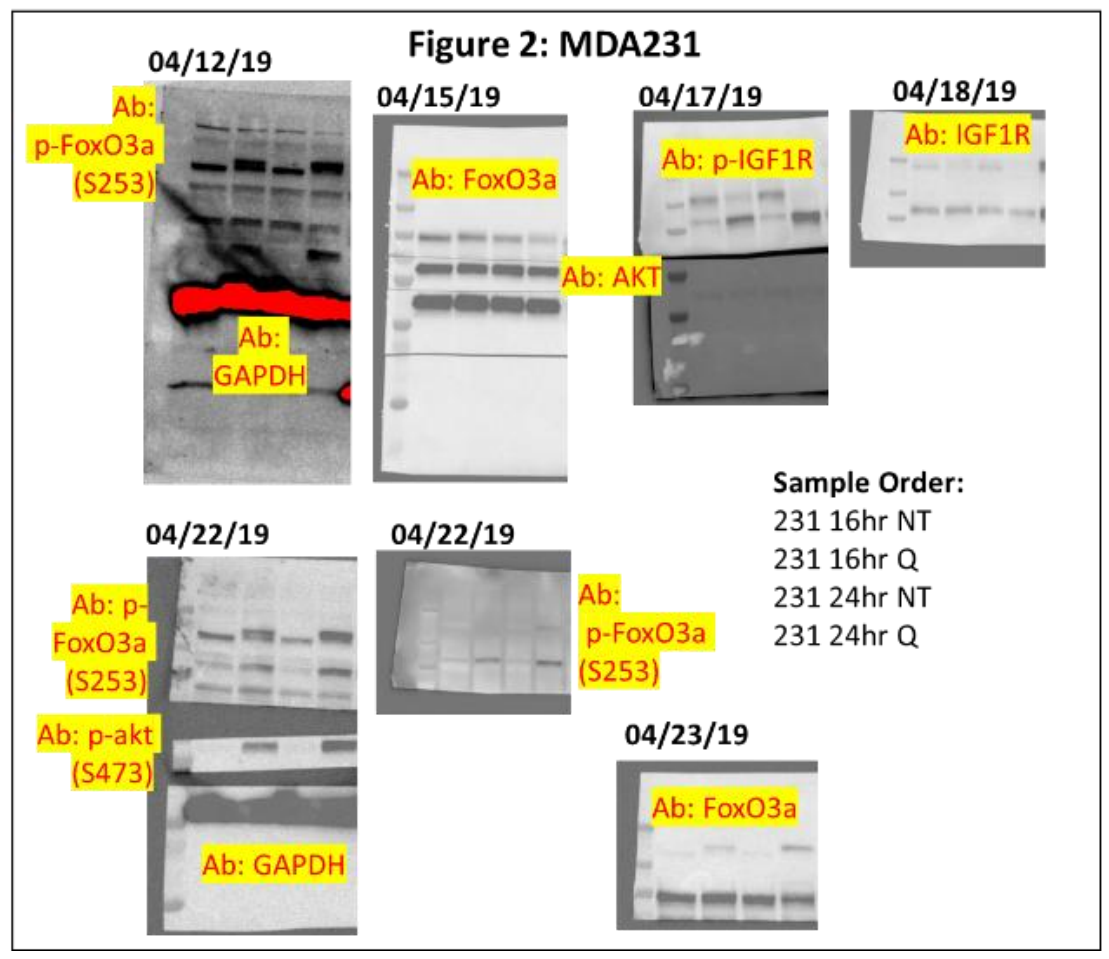

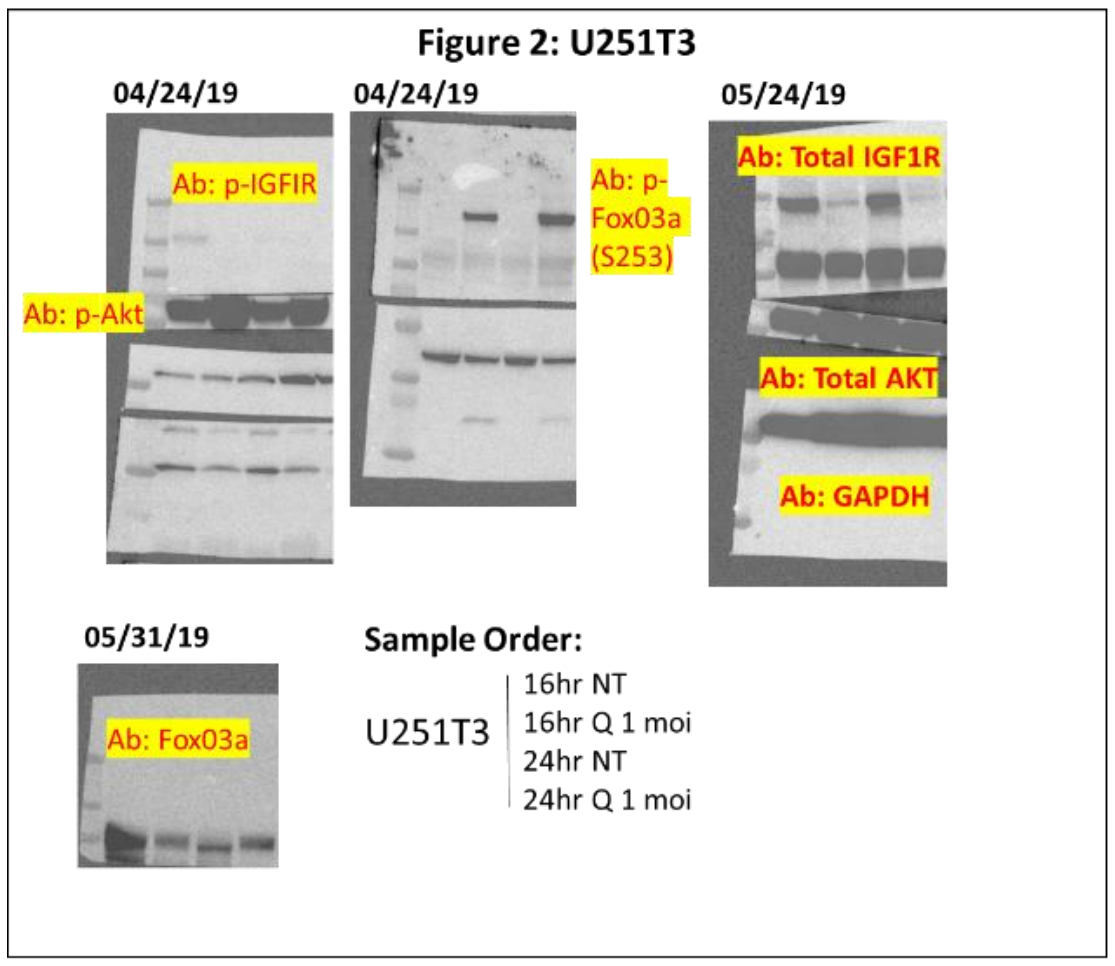
Figure 2: U251T3
04/24/19
04/24/19
05/24/19
Ab: p-IGFIR
Ab: p-Akt
Ab: p-Fox03a (S253)
Ab: Total IGF1R
Ab: Total AKT
Ab: GAPDH
05/31/19
Ab: Fox03a
Sample Order:
U251T3
16hr NT
16hr Q 1 moi
24hr NT
24hr Q 1 moi

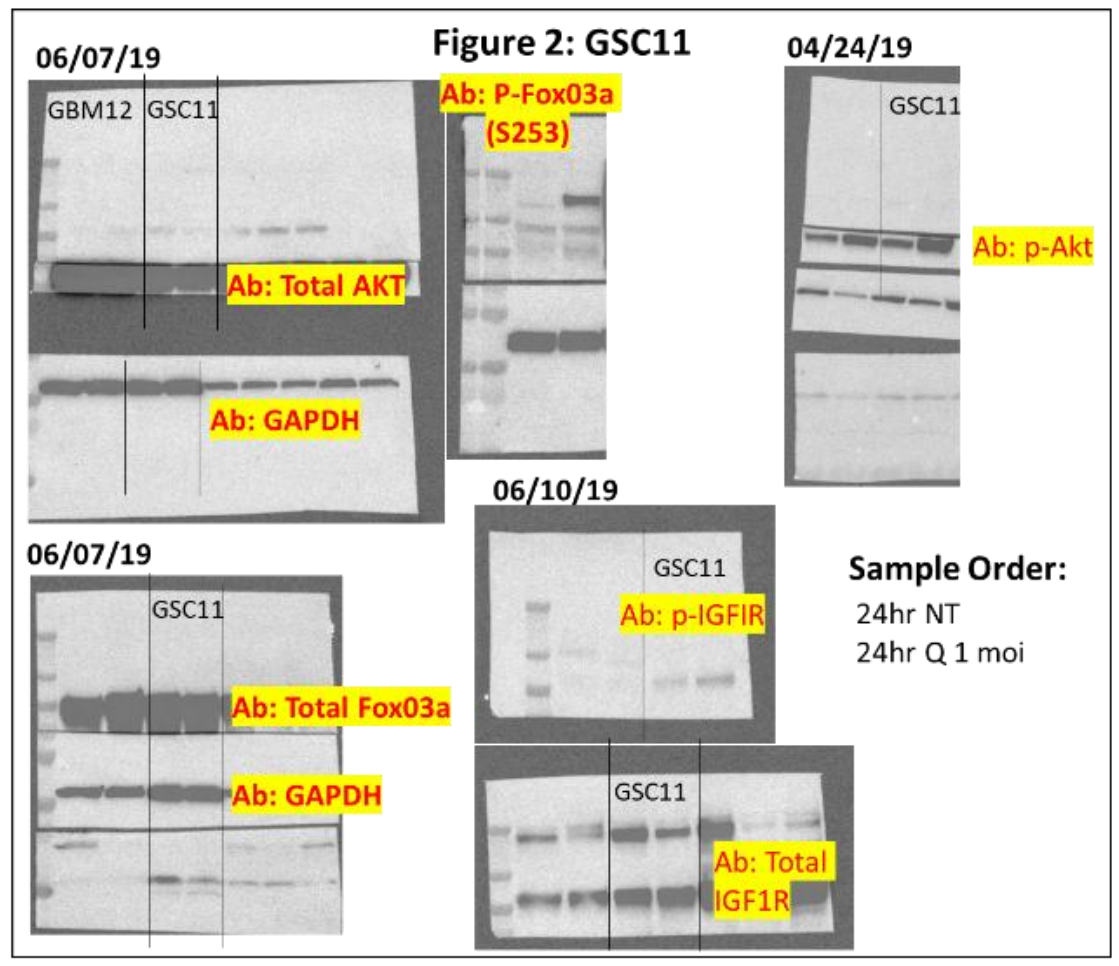
Figure 2: GSC11
06/07/19
GBM12
GSC11
Ab: Total AKT
Ab: GAPDH
Ab: P-Fox03a (S253)
04/24/19
GSC11
Ab: p-Akt
06/07/19
GSC11
Ab: Total Fox03a
Ab: GAPDH
06/10/19
GSC11
Ab: p-IGFIR
GSC11
Ab: Total IGF1R
Sample Order:
24hr NT
24hr Q 1 moi

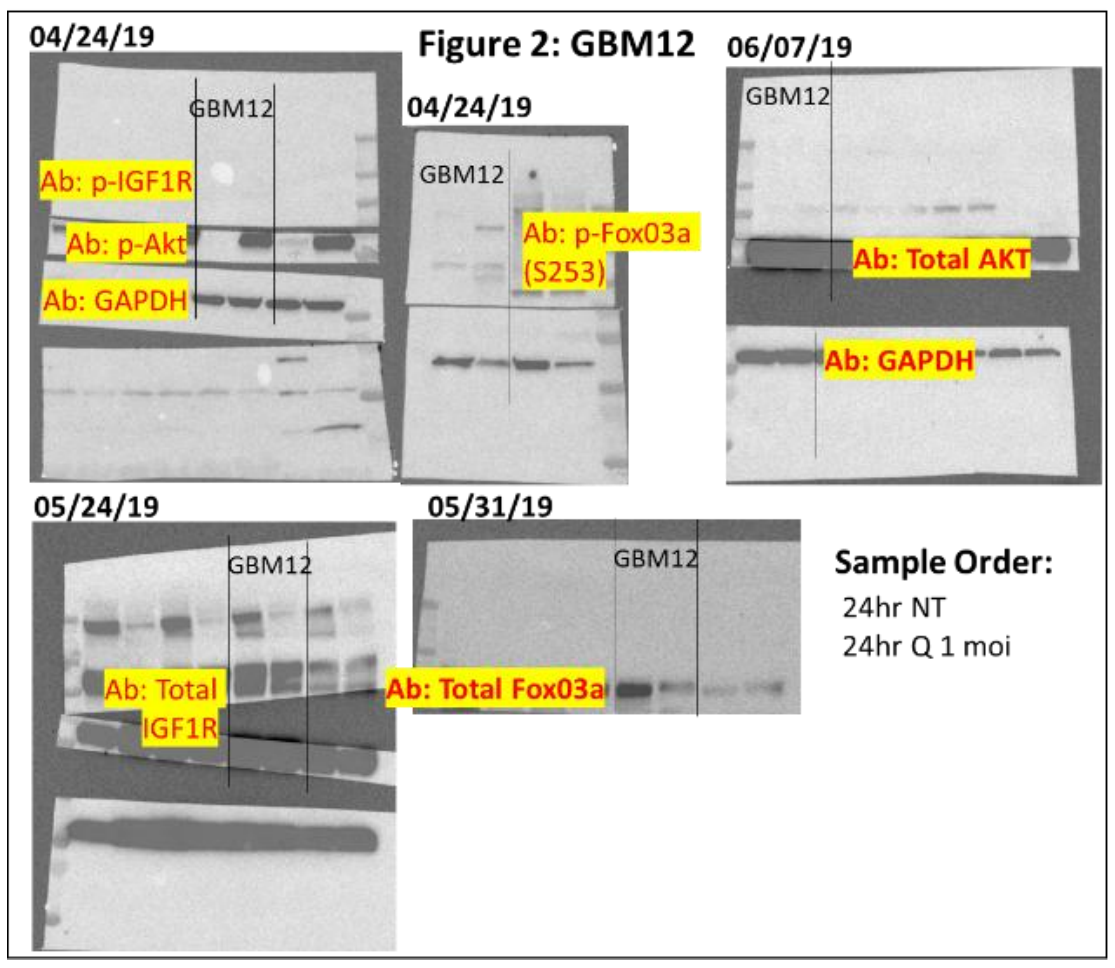
Figure 2: GBM12
04/24/19
GBM12
Ab: p-IGF1R
Ab: p-Akt
Ab: GAPDH
04/24/19
GBM12
Ab: p-Fox03a (S253)
06/07/19
GBM12
Ab: Total AKT
Ab: GAPDH
05/24/19
GBM12
Ab: Total IGF1R
05/31/19
GBM12
Ab: Total Fox03a
Sample Order:
24hr NT
24hr Q 1 moi

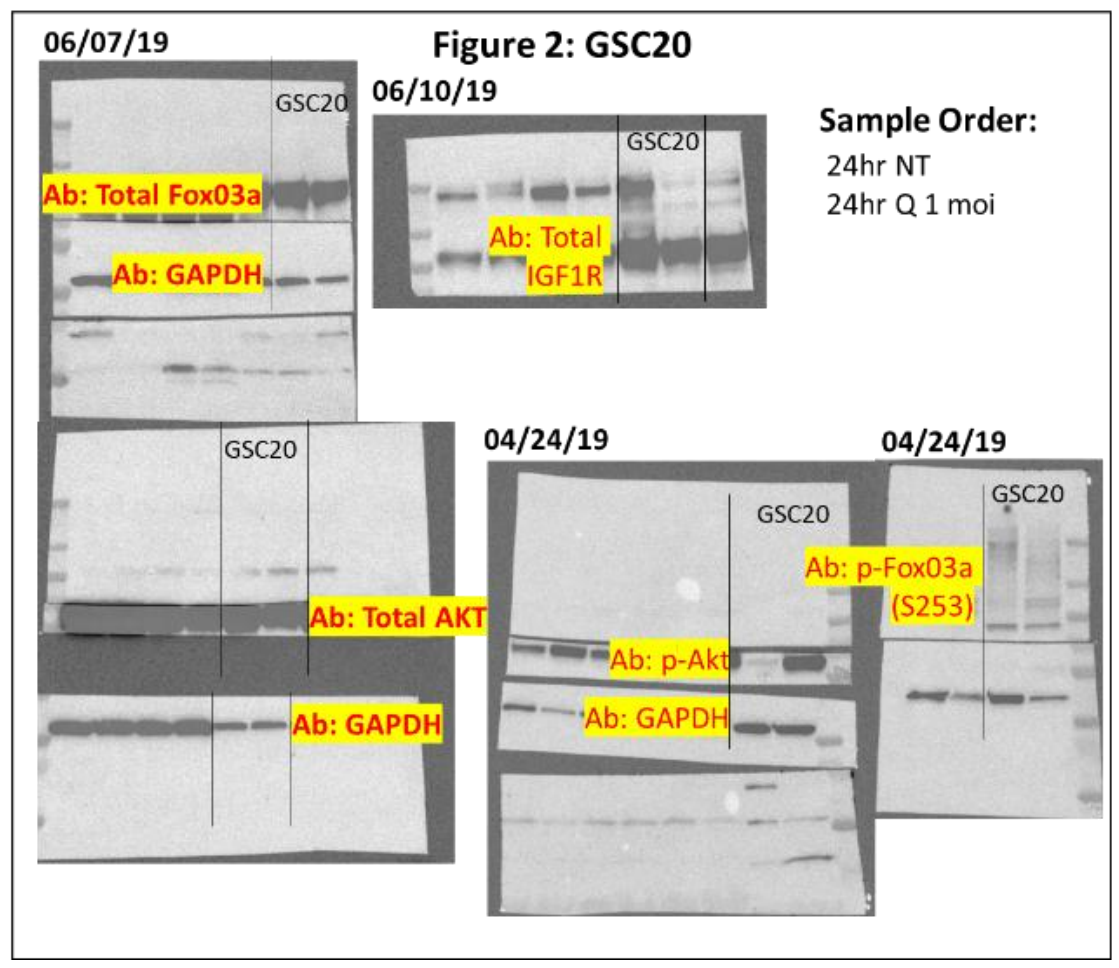
Figure 2: GSC20
06/07/19
GSC20
Ab: Total Fox03a
Ab: GAPDH
06/10/19
GSC20
Ab: Total IGF1R
Sample Order:
24hr NT
24hr Q 1 moi
GSC20
Ab: Total AKT
Ab: GAPDH
04/24/19
GSC20
Ab: p-Akt
Ab: GAPDH
04/24/19
GSC20
Ab: p-Fox03a (S253)

Figure 3: MCF7

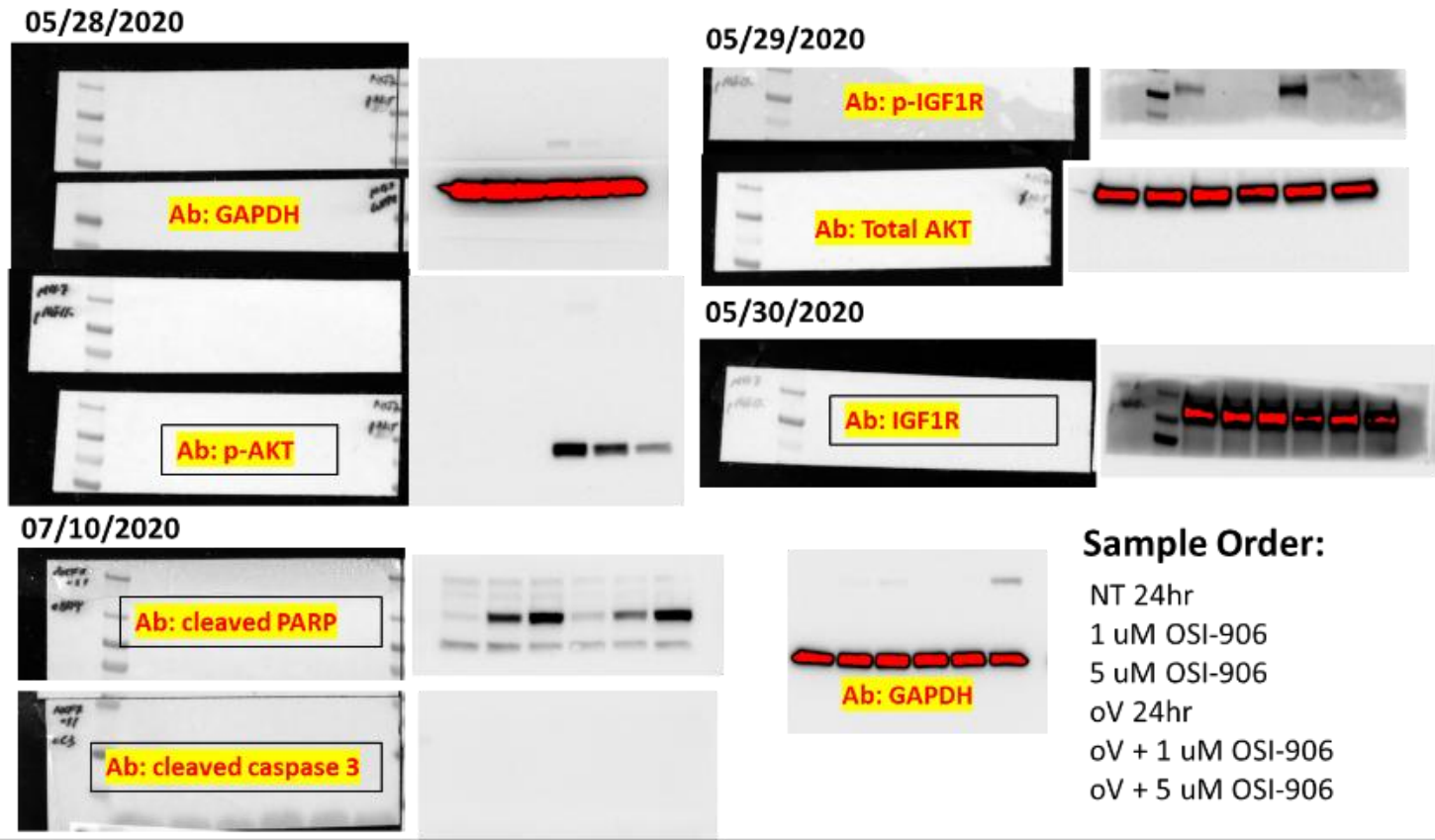

Sample Order:

NT 24hr
1 uM OSI-906
5 uM OSI-906
oV 24hr
oV + 1 uM OSI-906
oV + 5 uM OSI-906

Figure 3: SKBR3

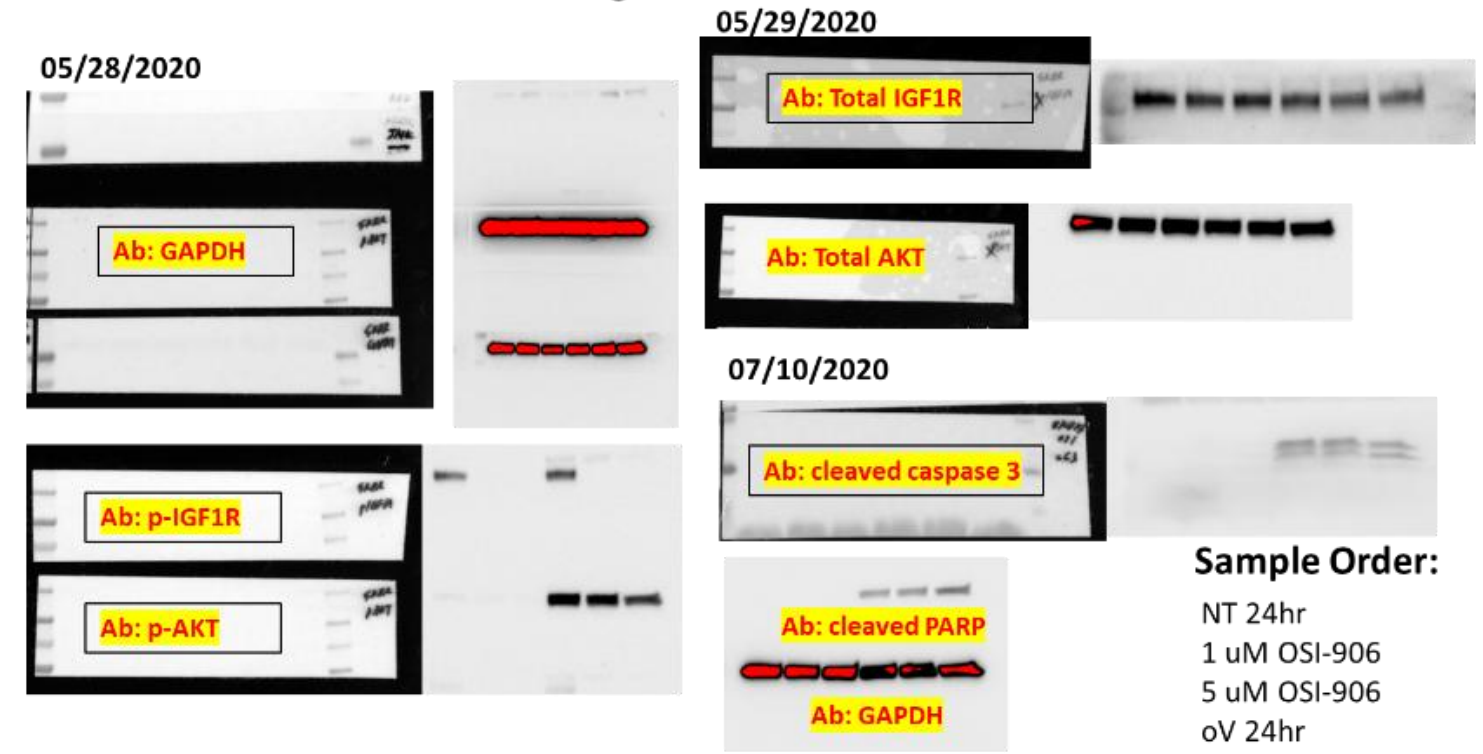

Sample Order:

NT 24hr
1 uM OSI-906
5 uM OSI-906
oV 24hr
oV + 1 uM OSI-906
oV + 5 uM OSI-906

Figure 3: DB7

Sample Order:

NT 24hr
1 uM OSI-906
5 uM OSI-906
oV 24hr
oV + 1 uM OSI-906
oV + 5 uM OSI-906

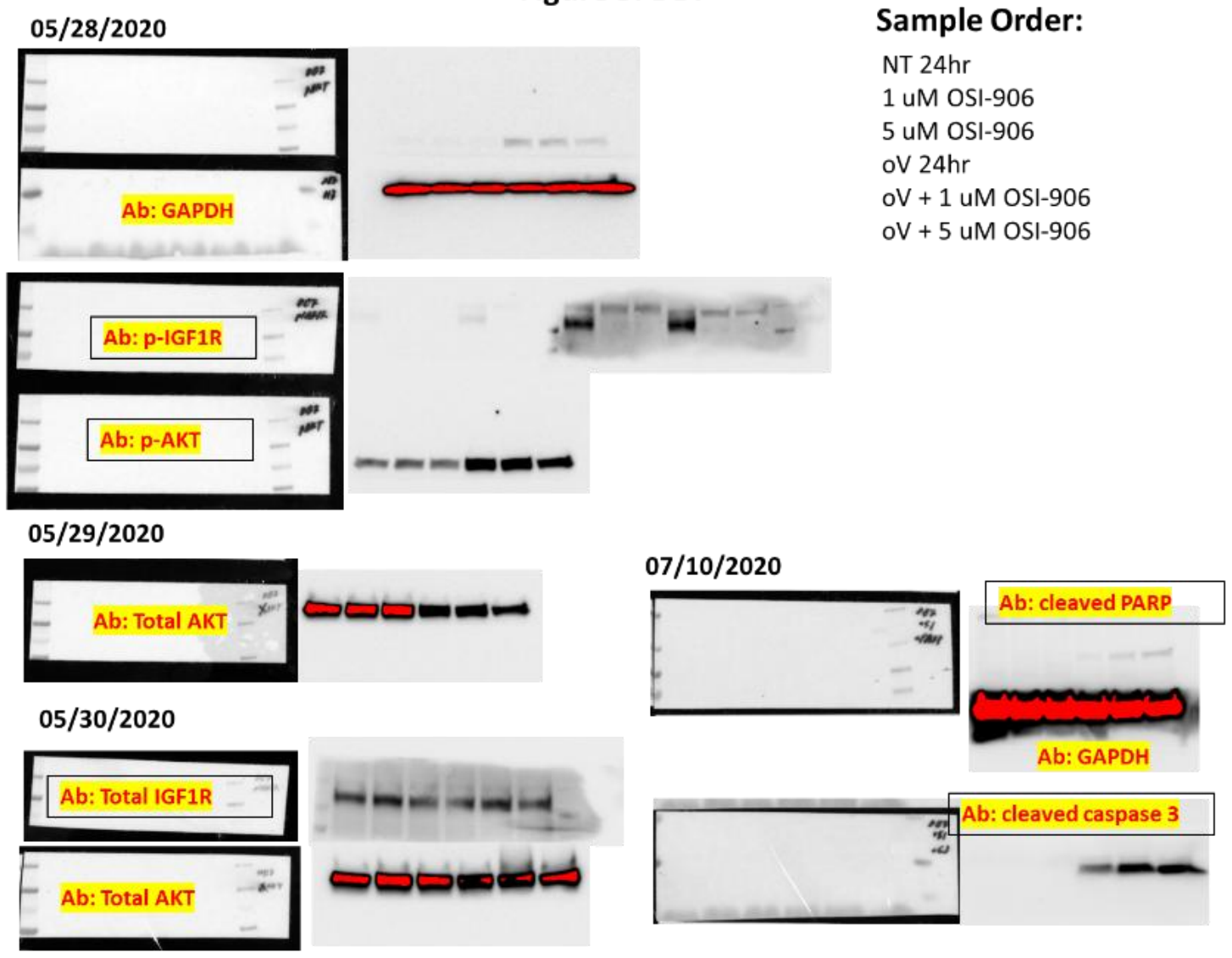

Figure 3: MDA468

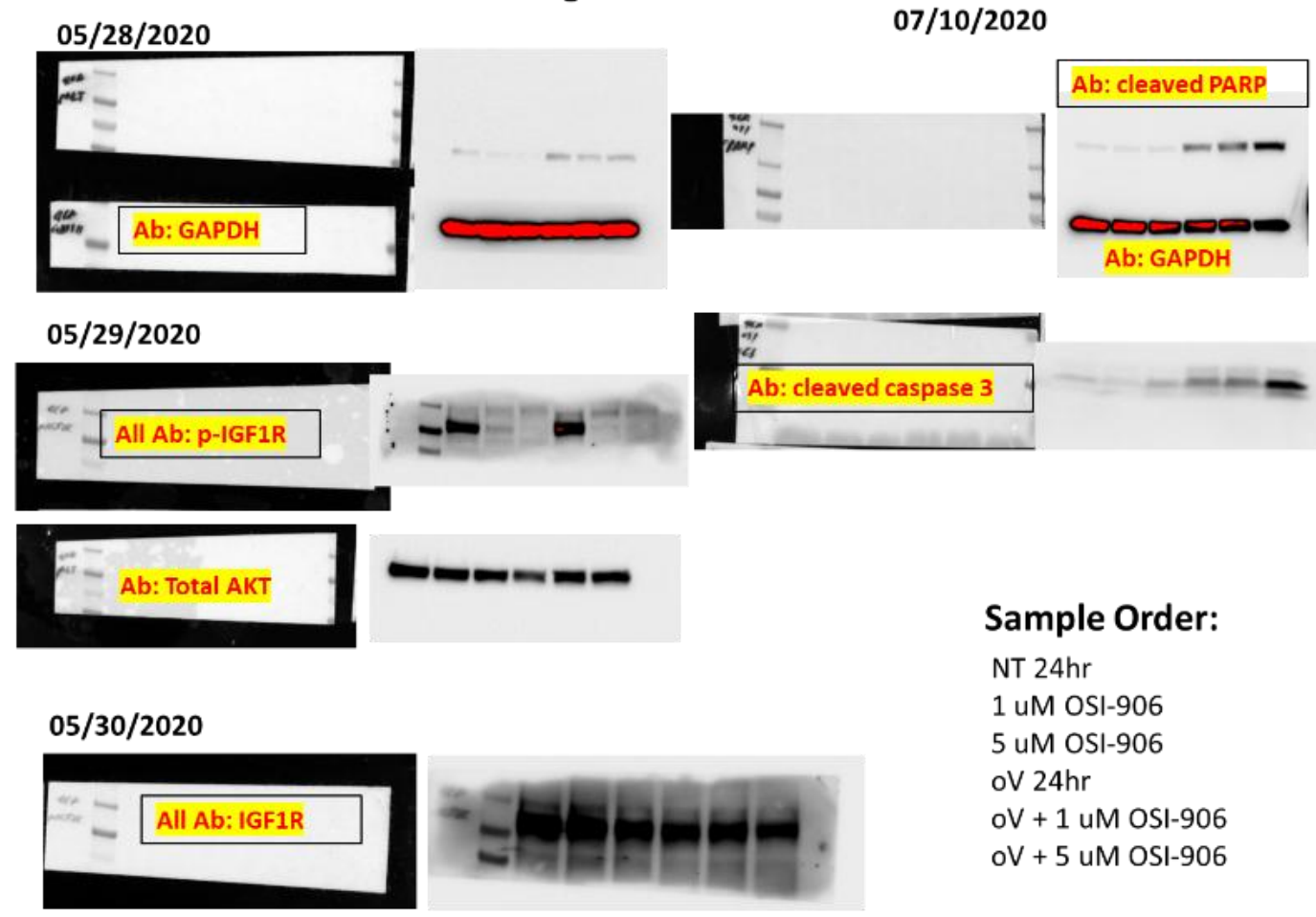

Sample Order:

NT 24hr
1 uM OSI-906
5 uM OSI-906
oV 24hr
oV + 1 uM OSI-906
oV + 5 uM OSI-906

Figure 3: GSC11

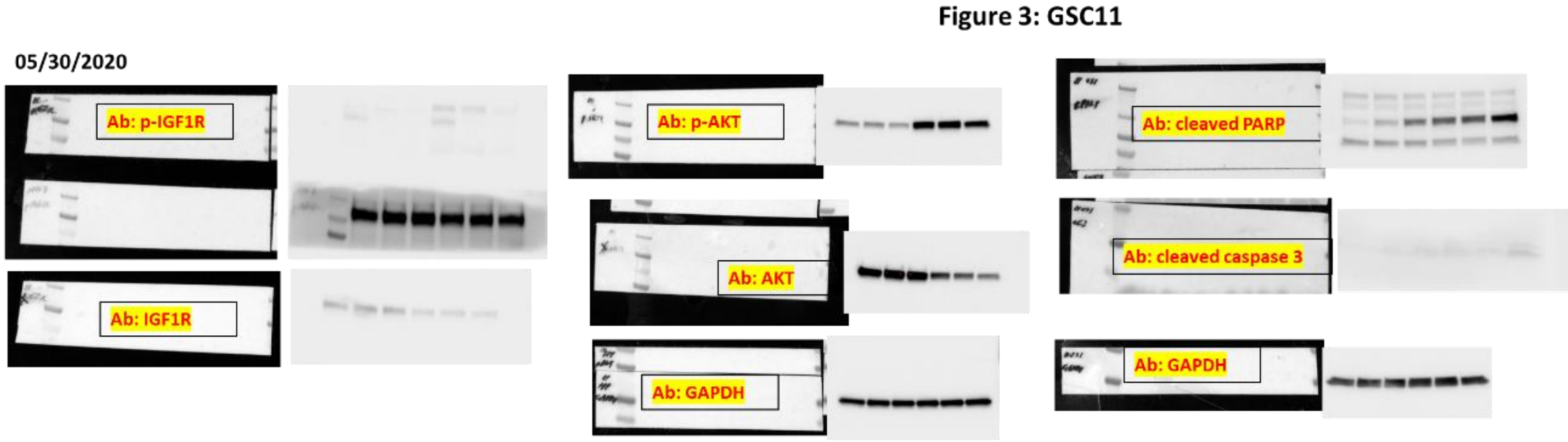

Sample Order:

NT 24hr
1 uM OSI-906
5 uM OSI-906
oV 24hr
oV + 1 uM OSI-906
oV + 5 uM OSI-906

Figure 3: MDA436 and GBM12

Western Blot by Jin Muk Kang 8/12/20

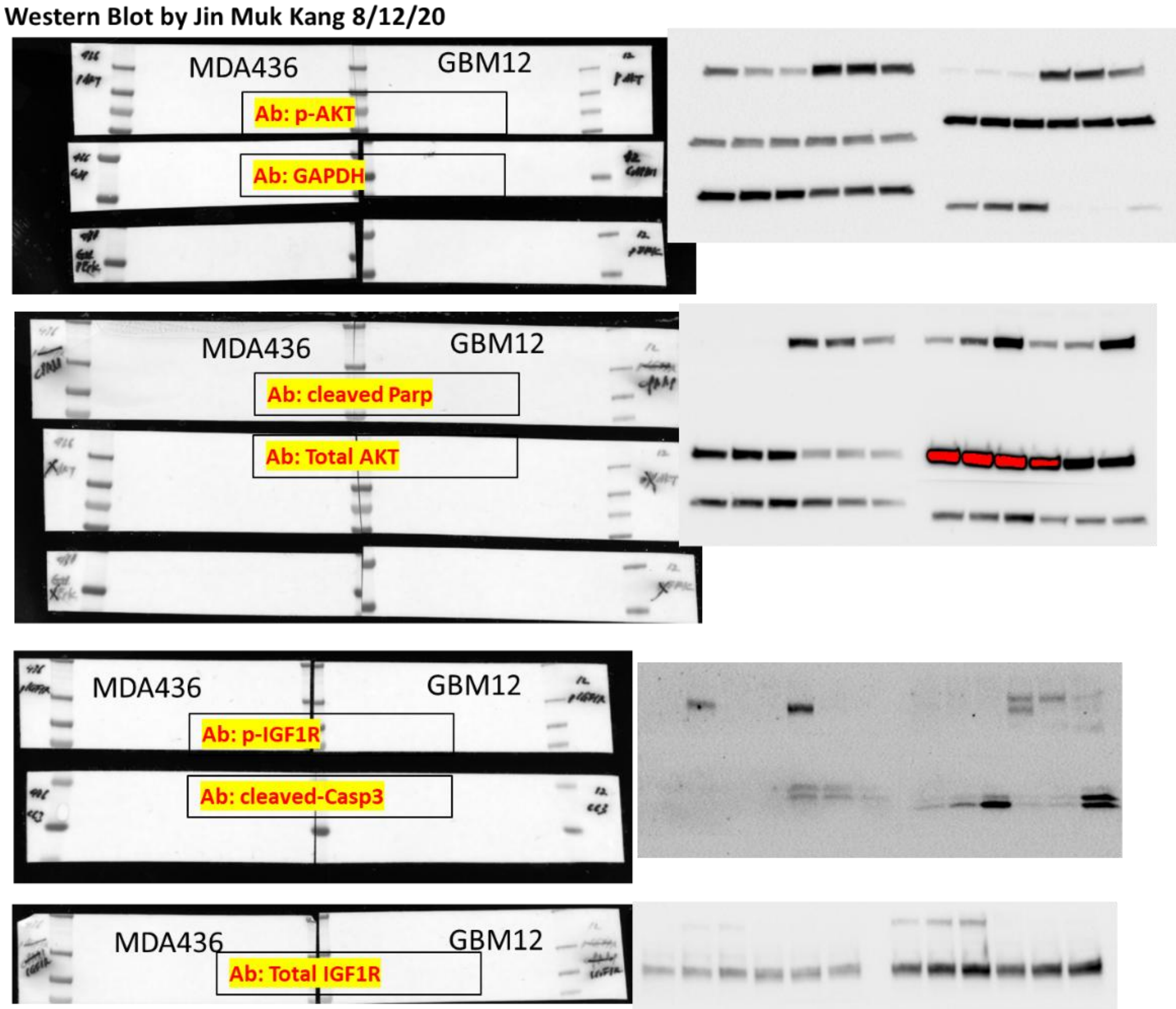

Sample Order:

All Cells
- NT 24hr
- 1 uM OSI-906
- 5 uM OSI-906
- oV 24hr
- oV + 1 uM OSI-906
- oV + 5 uM OSI-906

Figure 3: MDA231 and GBM6

Western Blot by Alexandra Miller 02/10/2026

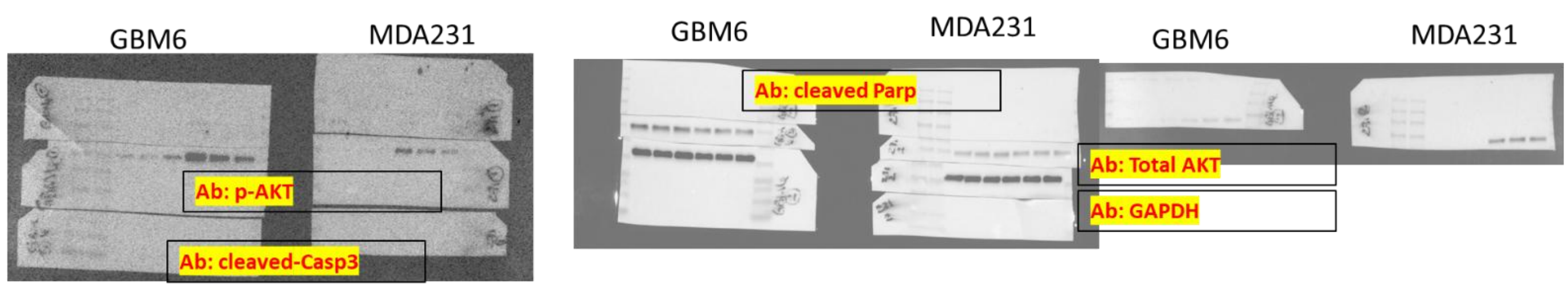

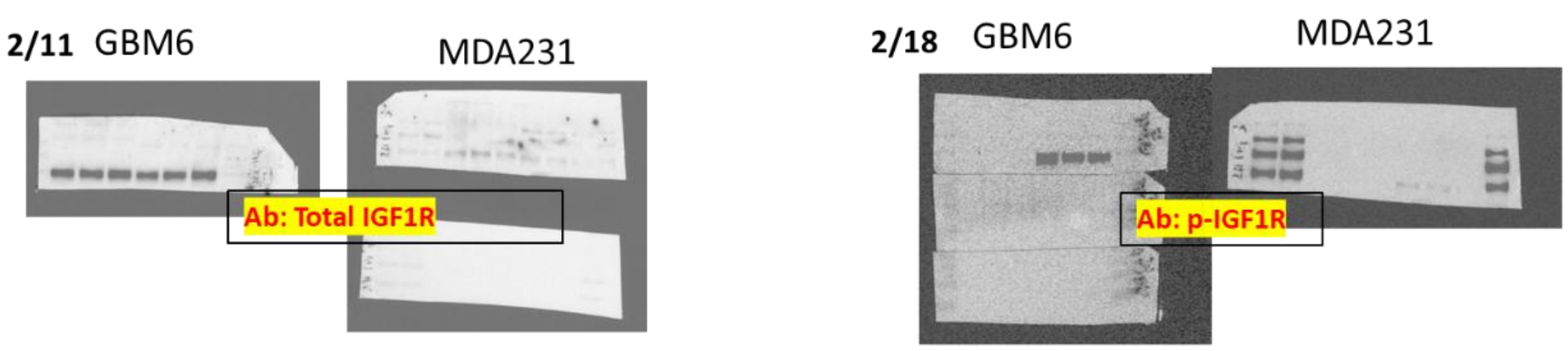

Sample Order:

All Cells
- NT 24hr
- 1 uM OSI-906
- 5 uM OSI-906
- oV 24hr
- oV + 1 uM OSI-906
- oV + 5 uM OSI-906

Figure 4: GBM6 and GBM28

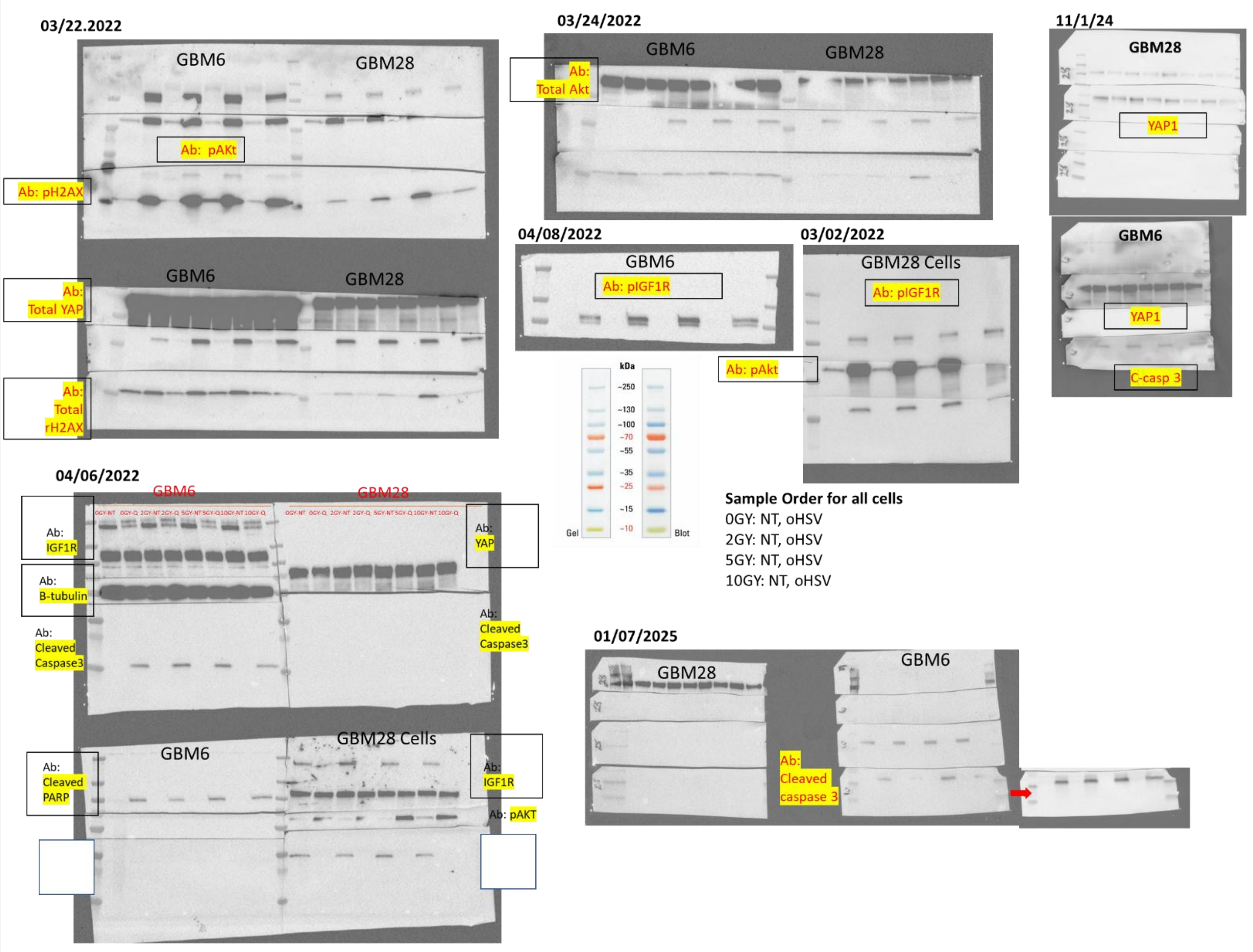

Figure 4: MDA468

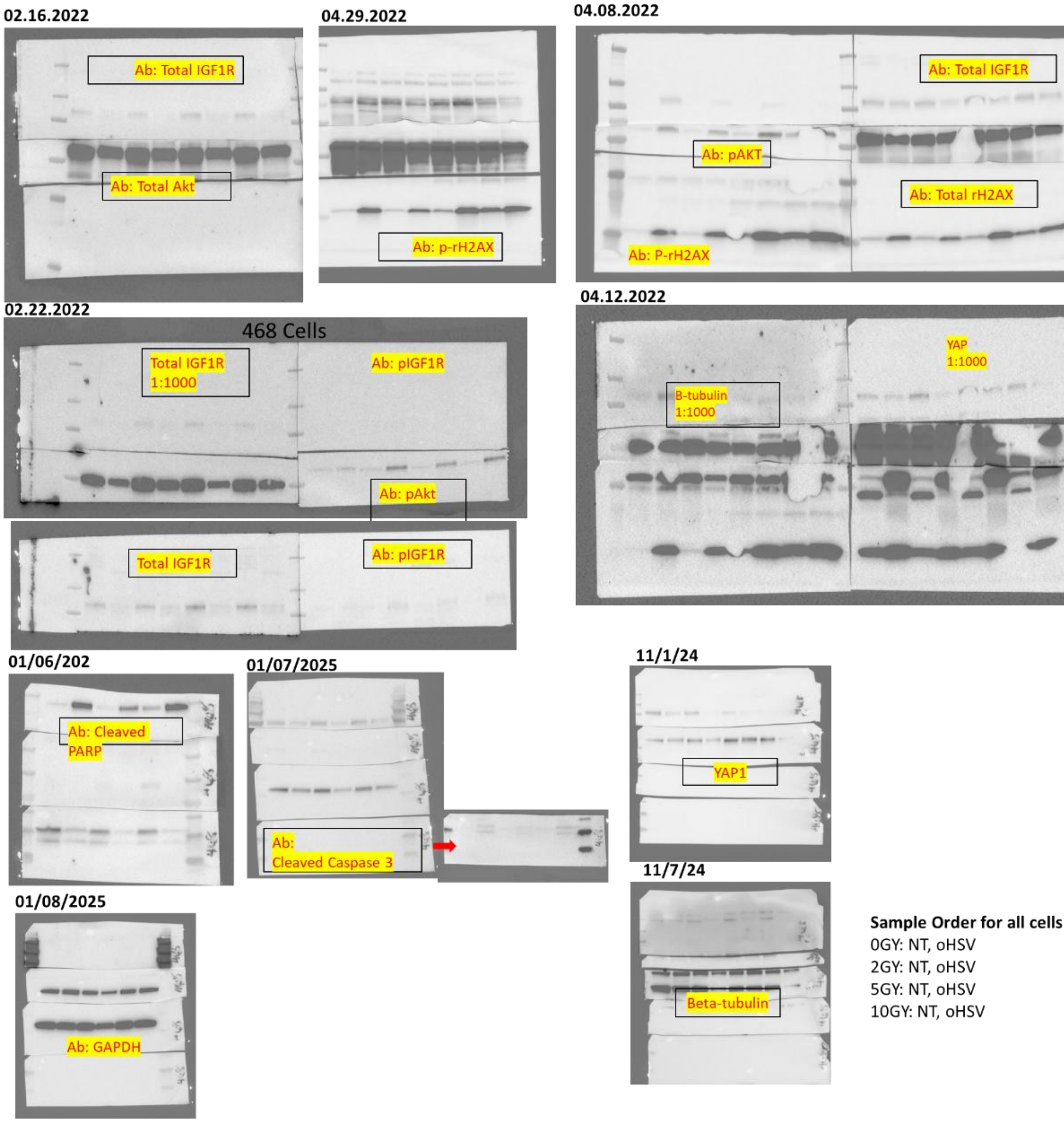

Figure 4: MDA436

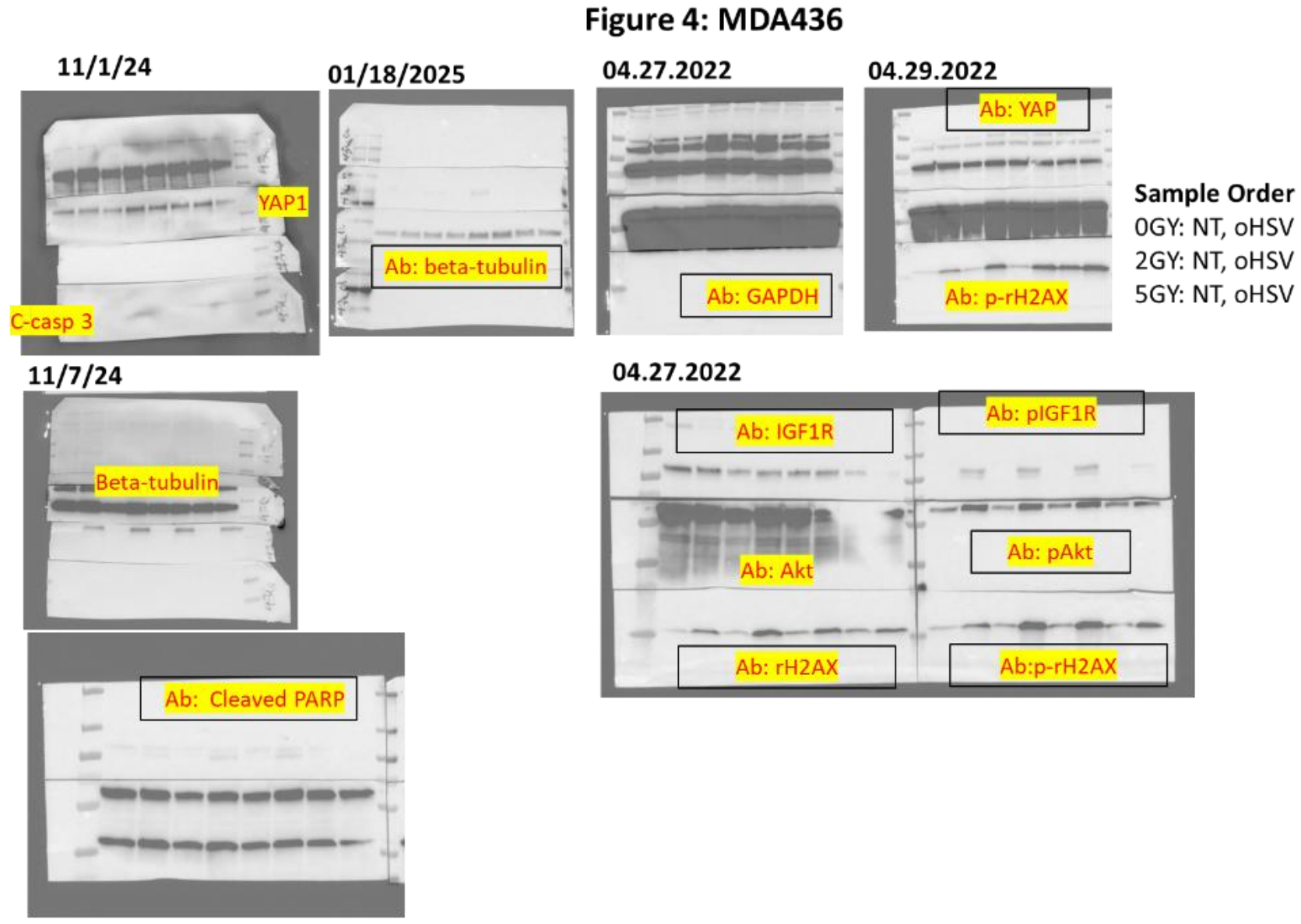

Figure 4: DB7

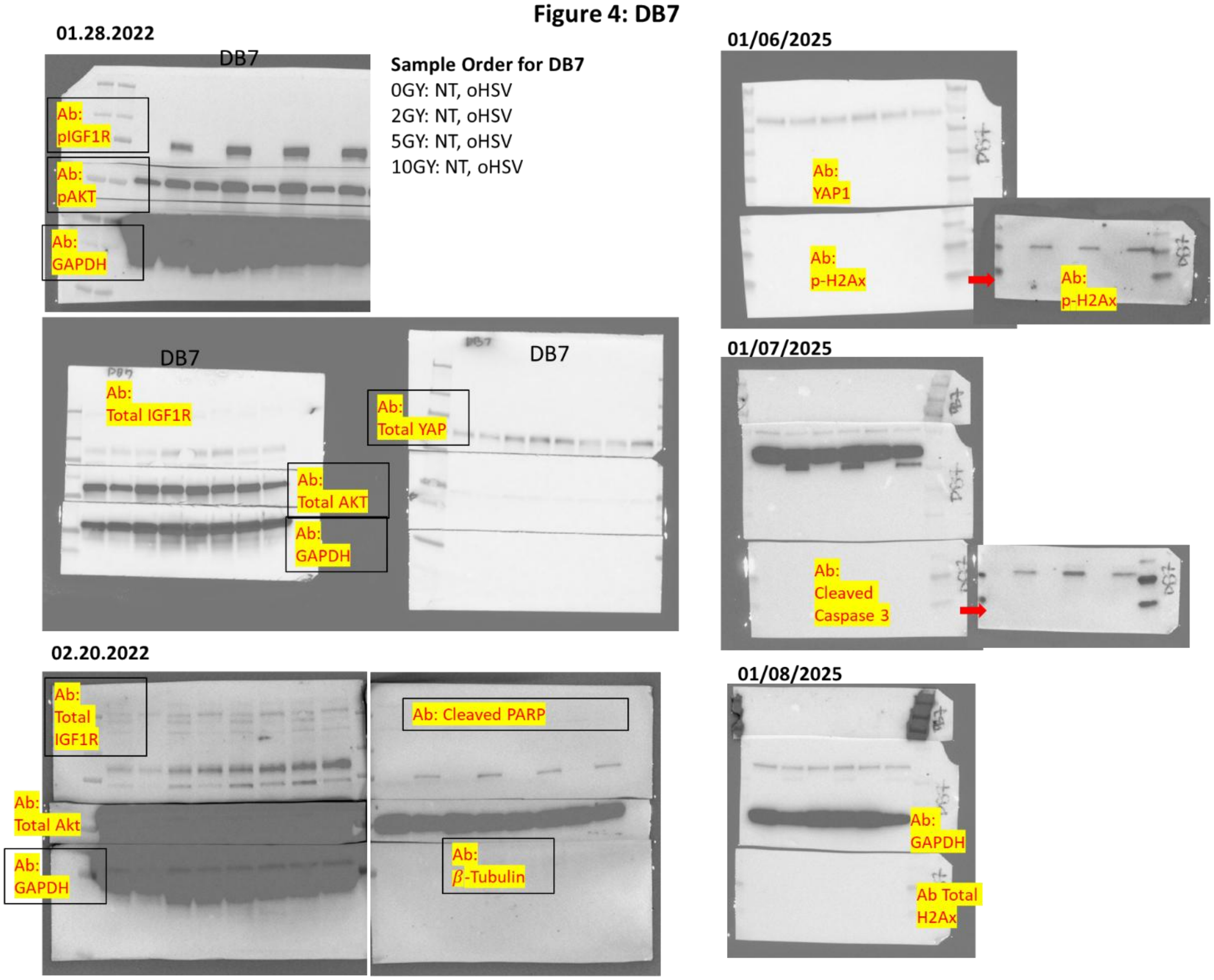

Figure 4: GBM12

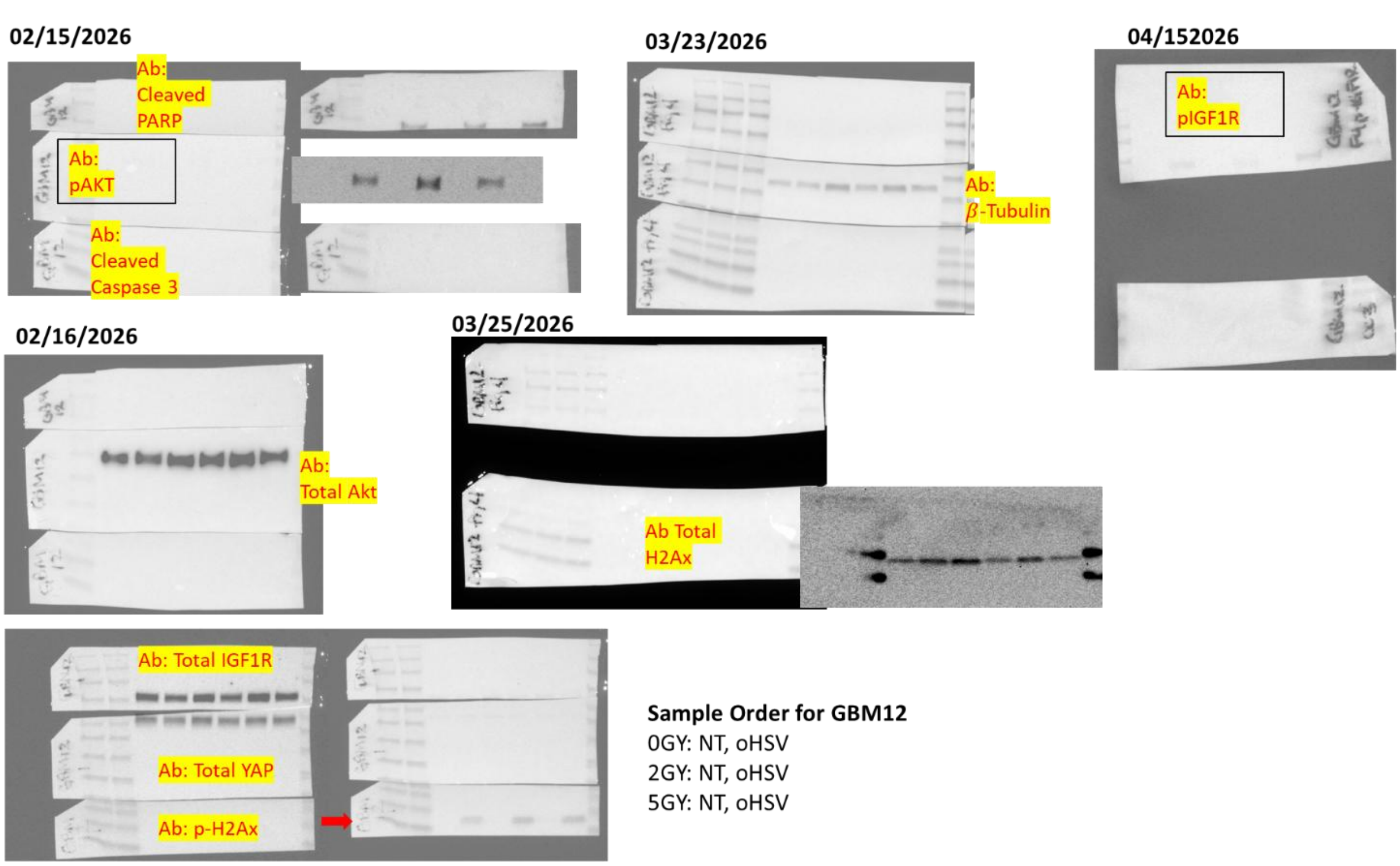

Figure 4: MDA231

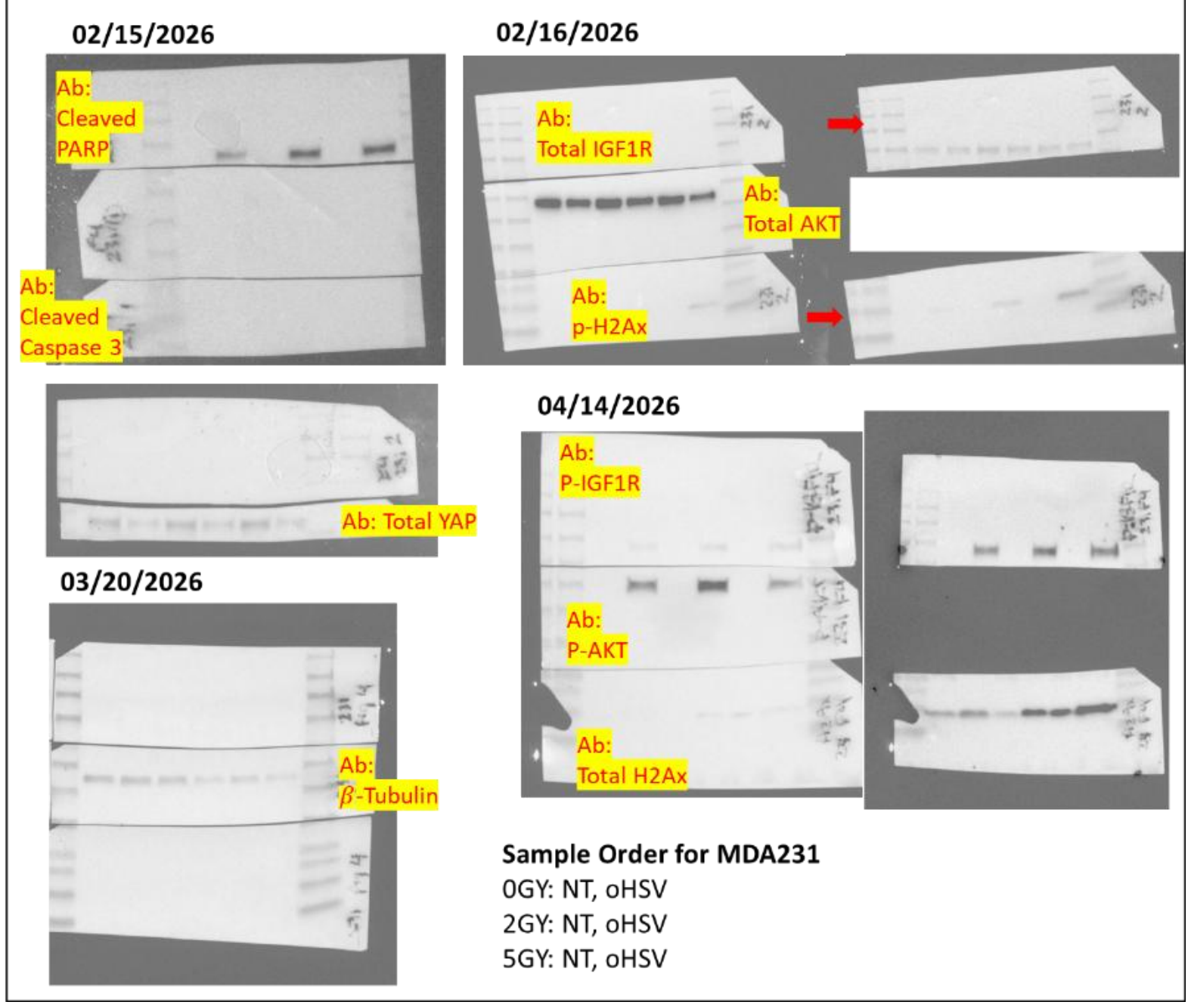

Figure 5: MDA468

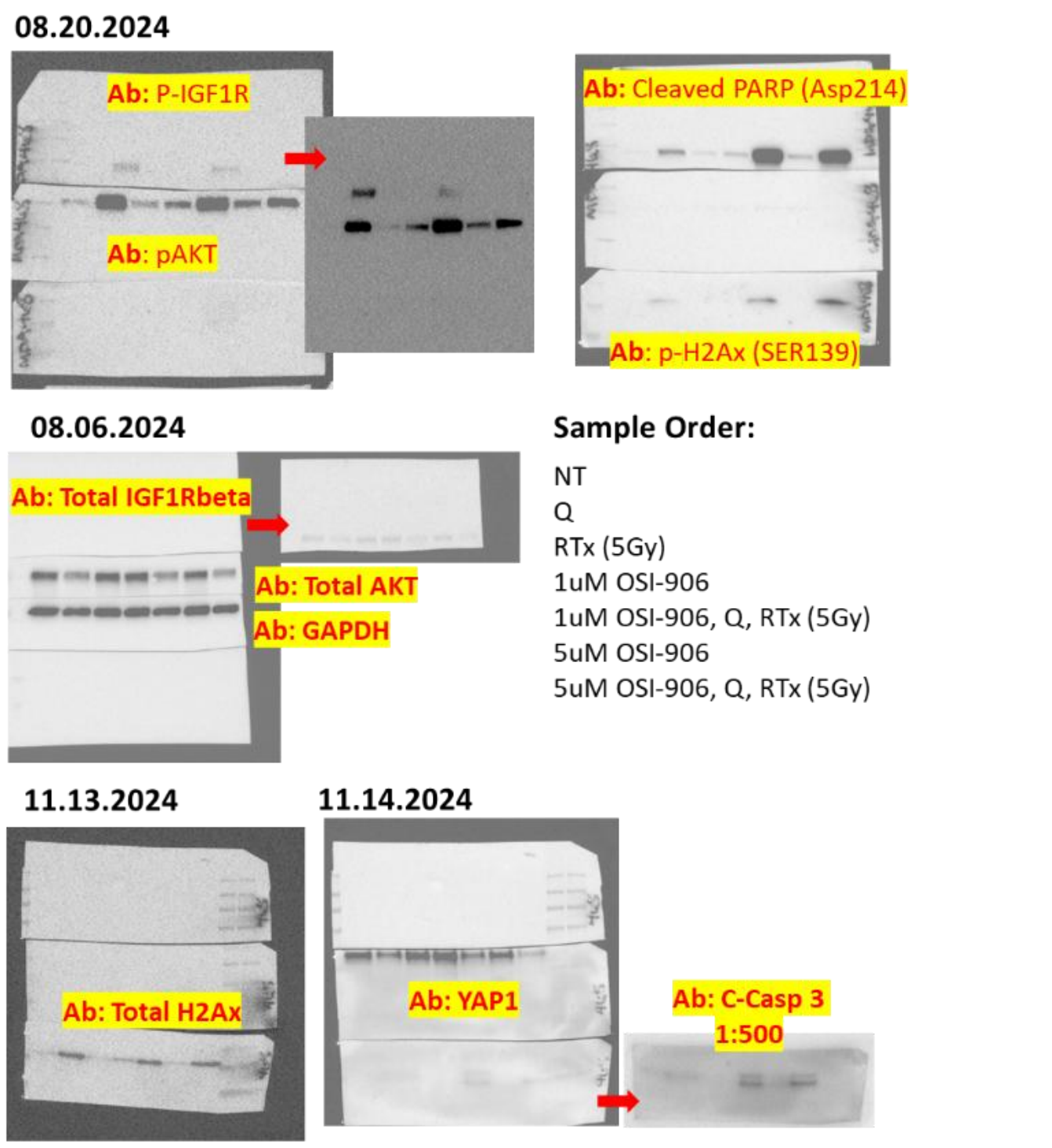

Figure 5: GBM6

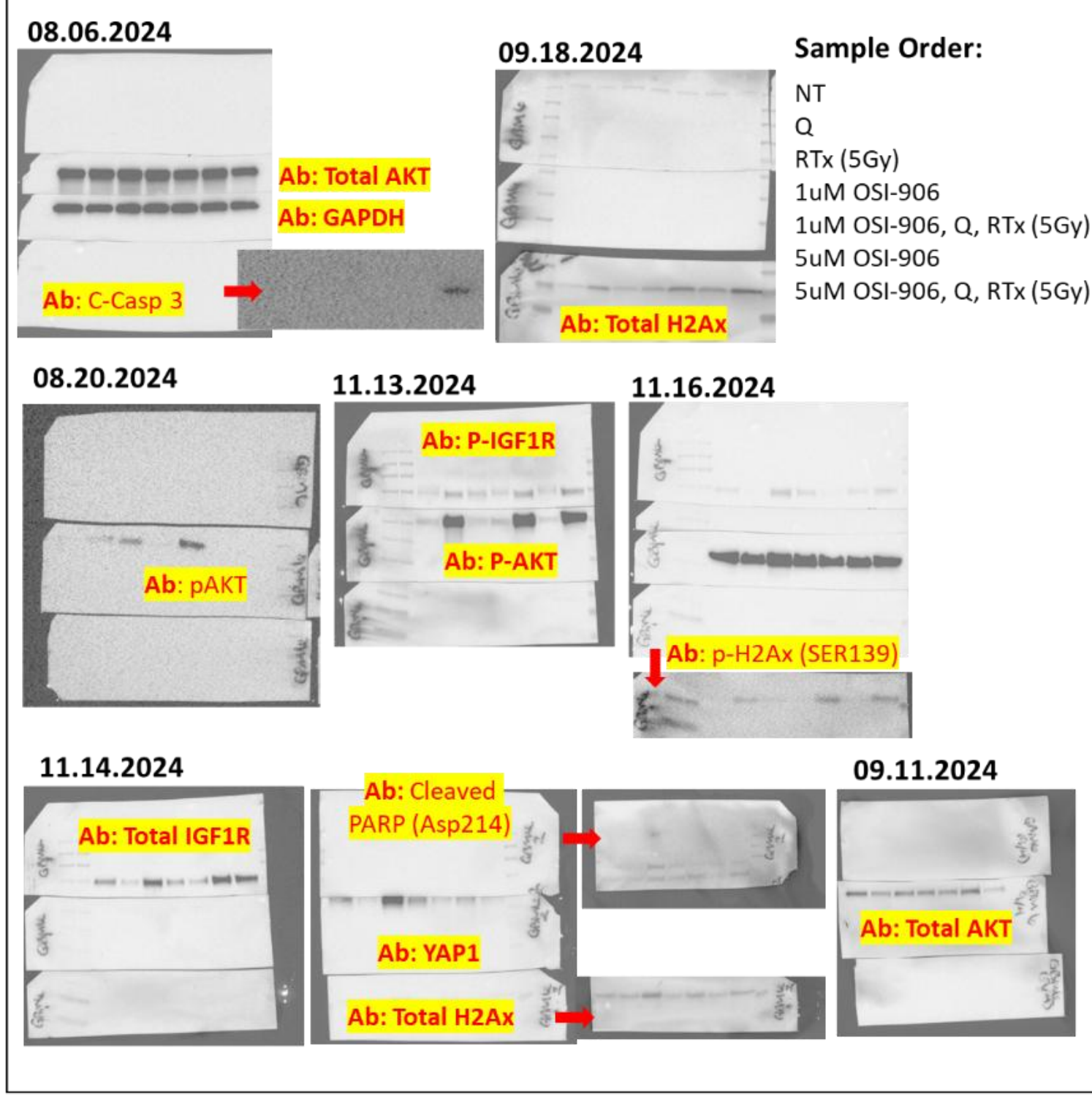

Figure 5: MDA231

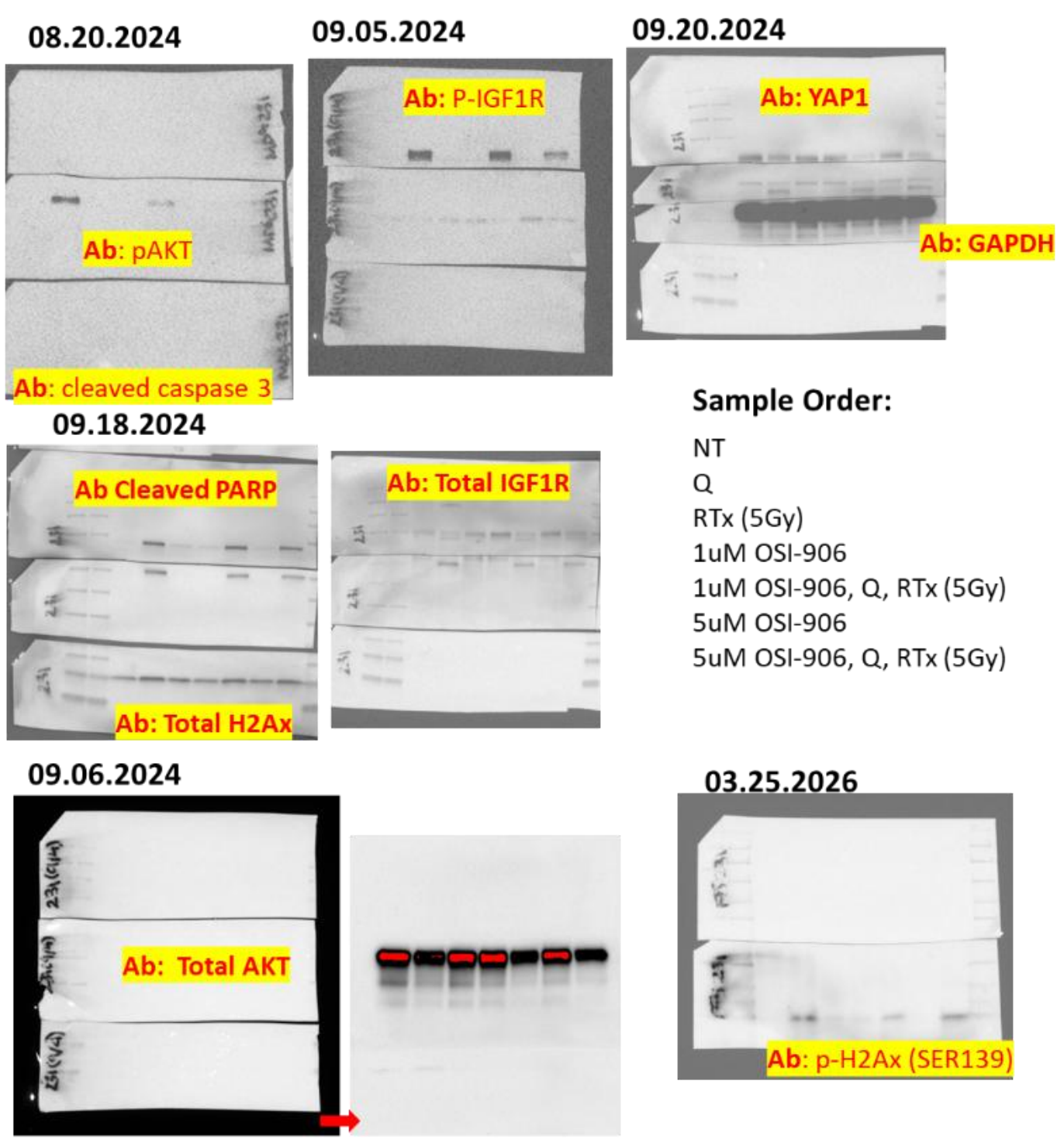

Figure 5: GBM12

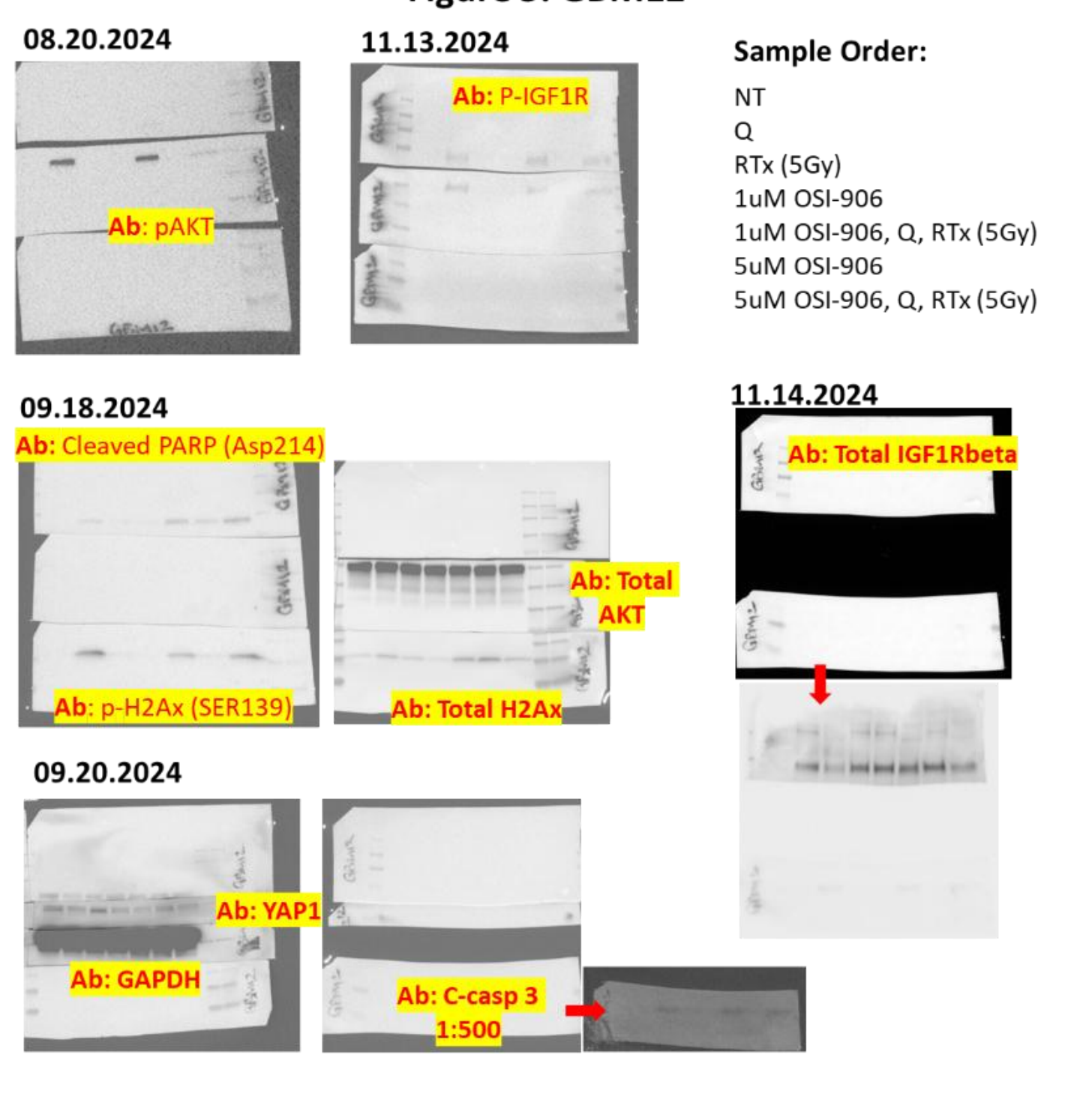

Supplement: Supplementary file 1 [file 41419_2026_9090_MOESM3_ESM.pdf]
