# Supplementary material for: Overcoming IGF1R-mediated resistance to oncolytic HSV1 and radiotherapy via triple combination therapy
Source: Cell Death Dis. 2026 Jul 17;17(1):881. doi: 10.1038/s41419-026-09090-5 (PMC13658246; doi:10.1038/s41419-026-09090-5)
Supplement: Supplementary file 2 [file 41419_2026_9090_MOESM1_ESM.docx]

**Overcoming IGF1R-Mediated Resistance to Oncolytic HSV1 and Radiotherapy via Triple Combination Therapy**

Alexandra A. Miller^1,2*^, Min-Hye Noh^1*^, Jin Muk Kang^1,3^, Jiyeon Kim^1^, Lily Nguyen^1,2^, Amanda S. Kouaho^1^, Grace Nguyen^1,4^, Minxin Huang^1,5^, Stephanie M. Bean^1^, Joshua Davis^1^, Matthew P Mullarkey^1^, Sunil Krishnan^1,2^, Zhongming Zhao^2,6^, E. Antonio Chiocca^7^, Tae Jin Lee^1,2#^, and Ji Young Yoo^1,2#^

^1^Department of Neurosurgery, McGovern Medical School, The University of Texas Health Science Center at Houston, Houston, TX, 77030, USA. ^2^The University of Texas MD Anderson Cancer Center UTHealth Houston Graduate School of Biomedical Sciences, Houston, TX 77225, USA. ^3^Department of Pediatric Hematology & Oncology, University Hospitals Cleveland Medical Center, Cleveland, OH, 44106, USA. ^4^Department of Pathology, University of Texas Southwestern Medical Center, Dallas, TX 75390, USA. ^5^Molecular and Cellular Pharmacology Program, Department of Pharmacological Sciences, Renaissance School of Medicine at Stony Brook University, Stony Brook, NY 11794, USA ^6^Center for Precision Health, McWilliams School of Biomedical Informatics, The University of Texas Health Science Center at Houston, Houston, TX 77030, USA. ^7^Harvey W. Cushing Neuro-oncology Laboratories, Department of Neurosurgery, Brigham and Women’s Hospital and Harvard Medical School, Boston, MA USA.

^*^These authors contributed equally.

**Supplementary Materials and Methods**

*Cell lines and Oncolytic Herpes Simples Virus-1 (oHSV-1)*

The patient-derived primary GBM cells, GBM6, GBM12, GBM22, GBM28, GBM39, and GBM43, were provided by Dr. Jann N. Sarkaria (Mayo Clinic, Rochester, MN) and GSC11 and GSC20 were obtained from MDAnderson Cancer Center (MDACC, Houston, TX). Primary GBM cells were maintained as tumor spheres in neurobasal medium supplemented with 1% B27 without vitamin A, human EGF (20 ng/mL), and basic FGF (20 ng/mL) in low-attachment cell culture flasks. DB7 (a murine breast cancer cell), MDA-MB-231, MDA-MB-436, and MDA-MB-468 human breast cancer cell lines were donated by the lab of Dr. Michael Ostrowski (The Ohio State University, Columbus, OH) and SK-BR-3 human breast cancer cells were gifted by the lab of Dr. Hui-Wen Lo (The University of Texas Health Science Center at Houston, TX). Cells are maintained in Dulbecco's Modified Eagle Medium–high glucose (DMEM; Corning, Corning, NY) supplemented with 10% fetal bovine serum (FBS). U251 cells were obtained from Dr. Erwin G. Van Meir (Emory University, Atlanta, GA), and U251T3 cells were created in our laboratory (May 2009) as a tumorigenic clone of U251 cells by serially passaging these cells three times in mice (PMID: 38853689). Vero cells derived from the kidney of an African green monkey, MCF7 human breast cancer cells, 4T1 murine breast cancer cells were purchased from the American Type Culture Collection (ATCC) and were maintained in DMEM supplemented with 10% FBS. Primary GBM12 (October 2018), GBM6, GBM28, and GBM39 (December 2021) were authenticated by the Cytogenetics and Cell Authentication Core at MD Anderson Cancer Center via short tandem repeat (STR) profiling. U251T3 (January 2015) cells were authenticated by the University of Arizona Genetics Core. All cells are routinely monitored for changes in morphology and growth rate. All cells were maintained below passage 40 and are negative for Mycoplasma contamination. For oncolytic HSV (oHSV), we used rHSVQ1, referred to as ‘oHSV,’ which contains a disruption in the UL39 locus and deletion of both copies of the γ34.5 gene (PMID: 38853689). This virus shares a similar backbone with CAN-3110, currently in clinical trials for high-grade brain tumors (NCT03152318), and with G207, which is also under investigation in brain tumor patients (NCT00028158). All viruses were propagated in Vero cells, and their respective titer (plaque-forming units per ml, PFU/ml) was quantified using the standard plaque-forming assay in Vero cells as previously described (Noh et al., 2024; Wakimoto et al., 2004).

*Western Blot Analysis and Antibodies*

Cells were lysed with RIPA buffer (GenDepot, Katy, TX) supplemented with protease and phosphatase inhibitor-100X (GenDepot). Cell lysates were fractionated by 4-20% Precast Midi Protein Gel (Biorad, Hercules, CA) and transferred to nitrocellulose (NC) blotting membrane (Genesee scientific, Morrisville, NC). The immunoreactive bands were visualized using an enhanced chemiluminescence (ECL) (GenDepot). Original full-length images of western blotting analysis are provided (Supplementary **Figure S16**). All antibodies used are listed in **Table S2**. Fig. 2 western blots were replicated by two researchers, Fig 3 western blots were replicated by three researchers, Fig. 4 western blots were replicated by two researchers, and Fig. 5 western blots were replicated by three different researchers.

*RNA Isolation, cDNA Synthesis, and qRT-PCR*

Total RNA was isolated using the RNeasy kit (Qiagen, Germantown, MD). cDNA was synthesized using the amfiRivert cDNA synthesis Platinum Master Mix kit (GenDEPOT, Katy, TX). qRT-PCR was then performed using the amfiSure qGreen Q-PCR Master Mix (2X), Low ROX (GenDEPOT) and detected using an Applied Biosystems QuantStudio 3 thermocycler. As internal expression control, we used 18S rRNA expression. Primers sequences were obtained from prior publications (indicated in **Table S2**) and were purchased from Integrated DNA Technologies (Coralville, Iowa) or Sigma-Aldrich (St. Louis, MO). The primer sequences used are described in **Supplementary Table S2**.

*Flow Cytometry*

For live/dead staining (Invitrogen, Waltham, MA), cells were harvested and subjected to incubation in 1ml of PBS containing 1ul of live/dead dye for 30min in the dark. The cells were then washed twice and resuspended in PBS. For Ki-67 staining, the cells were suspended in cold flow cytometry fixation buffer (BD Biosciences, San Jose, CA) and incubated at room temperature for 10 mins. The cells were washed twice with PBS and resuspended flow cytometry permeabilization/wash buffer I (R&D systems, Minneapolis,MN). Each treatment condition had three biological replicates that were then split into three technical replicates to assess variability introduced by the method itself. The Ki-67 antibody (Miltenyi Biotec, Bergisch Gladbach, Germany) was applied and incubated at room temperature in the dark for 30 min. The stained cells were analyzed using a Cytoflex (Beckman Coulter, Brea, CA). Single stain controls for compensation for each fluorochrome were prepared using cells or compensation beads (Invitrogen). Data was analyzed using FlowJo software. These data were replicated by three different researchers.

*Cell Proliferation Assay*

Primary GBM cells were plated in 96-well plates containing 1 x 10^4^ cells/well in 70µL of 2% FBS-containing media. The following day seeded cells were infected with oHSV at 5 MOIs prepared as a serial dilution by half in a volume of 15µL where the highest MOI was dependent on the innate viral sensitivity. One hour later, cells were treated with 0, 2, or 5Gy radiation using an RS 2000 Small Animal Irradiator (Rad Source Technologies, Inc.), then an hour later treated with DMSO, 0.05, 0.1, 0.5, 1 or 5µM OSI-906 by MedChemExpress LLC (Monmouth Junction, NJ, USA) in 15µl of 2% FBS-containing DMEM. Cells were incubated for 96 hours at 37℃, and cell viability was measured using a cell proliferation kit (Roche, Basel, Switzerland) following manufacturer’s protocol. Absorbance at 450nm was measured using a Synergy HT Multi-Mode Microplate reader (Biotek, Winooski, VT), and cell viability was measured as a percentage of uninfected untreated control. These data were replicated by four different researchers.

*Immunofluorescence, Immunocytochemistry, and Immunohistochemistry*

Mouse brains and mammary tumors were fixed in formalin for 24-48 hours then embedded in paraffin and sectioned by the UTHealth Histopathology Core. Representative IHC sections were stained with anti-phospho-IGF1R, anti-HSV-1, or anti-Ki-67 antibodies provided in **Table S2**. SignalStain Boost IHC Detection Reagent and DAB substrate kit (Cell Signaling Technology) were used and the sections were counterstained with hematoxylin. Representative IF sections and cells for ICC were stained with the primary and secondary antibodies provided in **Table S2** and then counterstained with Hoechst 33342 (Invitrogen). Imaging was obtained with Keyence (Itasca, IL, USA), EVOS-FL Auto2 (Invitrogen), or NIKON A1R-MP (NIKON, Tokyo, Japan). These data were replicated by three different researchers.

*Soft Agar Colony Forming Assay*

Cells were treated as described 24 hours prior to soft-agar colony formation assay. To assess anchorage-independent growth cells were prevented from adhering to the cell culture plate using a bottom agar layer prepared with the following final concentration: 0.8% agarose (Promega, Madison, WI) in 10% FBS in 0.5X DMEM, pre-warmed at 37ºC, transferred to clear flat-bottomed 6-well plates, and chilled at 25ºC to solidify. Cells were harvested and dissociated into single-cell suspensions using 0.25% trypsin-EDTA solution (GeneDEPOT). Cell number was determined as an average of three counts, then adjusted to the targeted cell density by diluting with 5% FBS and GlutaMAX (Gibco, Grand Island, NY, USA) in DMEM and mixed into 1.4% agar at a ratio of 1:1 to obtain a final concentration of 0.7% agarose in 2.5% FBS, 1.5X GlutaMAX in 0.5X DMEM. For each well, 1ml of the 0.7% agarose containing cells was layered on top of the bottom agar and solidified at 25ºC for 30 minutes. Each condition was performed in triplicates. After complete solidification, 500ul of 10% FBS DMEM was overlaid on the top, changed every 5-6 days, and cells were cultured at 37ºC in 5% CO2 for 14-30 days (Franken et al., 2006). These data were replicated by two researchers.

*Soft Agar Colony Forming Assay Staining and Analysis*

At the end of the experiment, triplicate images of colony shapes and sizes were taken for each well at 4X magnification for colony number and shape quantification using ImageJ software (NIH, USA). Colonies were then fixed with 2% paraformaldehyde for 30 minutes, then stained with 0.0005% Crystal Violet for 30 minutes. Tumorspheres were then washed with diH_2_O until colonies were clearly distinguishable from agar (5 minutes) and high-resolution images were taken. Total well area covered and intensity of tumor-sphere staining was determined using the ColonyArea Plugin for ImageJ as described (Guzmán et al., 2014).

*Animal Studies*

All mouse housing and experiments were performed in accordance with the Animal Welfare Committee at the University of Texas Health Science Center in Houston guidelines and have been approved by the Institutional Review Board. Animal studies were not double-blinded. In the case of mammary fatpad and subcutaneous GBM tumor models, all animals were implanted with tumors, which were allowed to grow for the amount of time indicated in the figure legend and treatment schedule graphic (7-10 days) then were distributed across treatment groups such that average initial tumor sizes were even. In the case of intracranial models of GBM, tumor size was unknown at the time of treatment, and were thus randomly assigned to each treatment arm.

Six- to eight-week-old NSG, FvB/n, athymic nu/nu, and Balb/C mice were obtained from Jackson Laboratory (Bar Harbor, ME, USA). For intracranial tumor studies, anesthetized mice were fixed in a stereotactic apparatus, and a burr hole was drilled at 2 mm lateral and 1 mm front to the bregma, to a depth of 3.5 mm. 1 x 10^5^ GBM12 and GBM28 human primary GBM cells into NSG mice and 1 x 10^5^ DB7 murine breast cancer cells into FvB/n mice were implanted intracranially. Ten days post tumor cell implantation, mice were randomized and injected intratumorally with PBS or 5 x10^5^ pfu of oHSV. 11 and 13 days post-tumor implantation, mice were anesthetized and irradiated (2Gy) using the RS 2000 Small Animal Irradiator (Rad Source Technologies, Inc.). 25mg/kg PPP (Cat. No. S7668, Selleck Chemicals, Houston, Texas, USA) was then administered by oral gavage 5 days per week for 4 weeks. For survival studies, animals were observed daily and euthanized at the indicated time points or when they showed signs of morbidity (hunched posture and/or weight loss or visible necrosis). 1 x 10^6^ MDA-MB-231 human breast cancer cells were injected into the mammary fat pad of female nu/nu mice and 4 x 10^5^ 4T1 murine breast cancer cells into Balb/C mice, followed by 14 days post-injections of PBS or 5 x 10^5^ pfu of oHSV into the same location by intraperitoneal injection. As in our intracranial models, mice were irradiated (2Gy) 11 and 13 days post-treatment followed by oral administration of 25mg/kg OSI-906 (MedChemExpress LLC) 5 days per week for 4 weeks. The drug was solubilized in 45% saline, 40% PEG-300, 10% DMSO, and 5% Tween-80. The tumor volume (mm^3^) was measured every 2 days after injections of PBS or oHSV and calculated using the equation: Tumor volume = length x (width)^2^ x 0.5. For histologic and immunofluorescence studies, mice were euthanized and tissue collected 2 or 7 days post-treatment.

*Statistical Analysis*

Statistical analyses were performed using GraphPad Prism version 10 (GraphPad, San Diego, CA, RRID:SCR_002798)) or Python (Version 3.13). Unpaired two-sided student’s t-test with Welch’s correction or Mann-Whitney U test was used to test the difference in comparison of continuous data between the two groups. A two-stage step-up method of Benjamini, Krieger, and Yekutieli false discovery approach was used with the desired FDR (Q) threshold of 0.01 (1%) (Benjamini et al., 2006). To analyze survival data, Kaplan-Meier curves were compared using the log-rank test and the post hoc pairwise groups test (if applicable) was further performed by Benjamini and Hochberg correction. *P* values were adjusted for multiple comparisons by Holms’ procedure. Individual two-sided unpaired t-tests and two-way ANOVAs were additionally performed to compare two treatment conditions at a time. A *p* value less than 0.05 was considered statistically significant. All statistical findings are summarized in the **Supplemental Statistics**. Of note, no samples were excluded from the experiments performed in this study.

Dose-response curves, synergy and sensitivity (Malyutina et al., 2019) calculations and interaction landscapes were made using SynergyFinder+ (Zheng et al., 2022). Synergy was calculated using the HSA, Bliss Independence (Bliss, 1939), Loewe (Loewe, 1953), and Zero Interaction Potency (ZIP) models (Yadav et al., 2015) sensitization was measured by the combination sensitivity score (CSS) and relevant inhibition (RI) summarized in **Supplementary Table S3**.

**Supplementary Dat****a**

**Supplementary Table S1. Antibodies used in this study.**

**Supplementary Table S2. RT-qPCR primers used in this study**

| **Host Specimen** | **Gene** | **Type** | **Sequence** | **Source Citation** |
| --- | --- | --- | --- | --- |
| Homo sapiens | CCN1 | Forward | 5'-AATGGAGCCTCGCATCCTATA-3' | (Kurozumi et al., 2008) |
|  |  | Reverse | 5'-TTCTTTCACAAGGCGGCA-3' |  |
|  | CCN2 | Forward | 5'-CTTGCGAAGCTGACCTGGAAGA-3' | ORIGENE |
|  |  | Reverse | 5'-CCGTCGGTACATACTCCACAGA-3' |  |
|  | 18S rRNA | Forward | 5'-GAGGGAGCCTGAGAAACGG-3' | (Noh et al., 2024) |
|  |  | Reverse | 5'-GTCGGGAGTGGGTAATTTGC-3' |  |

**Supplementary Table S3. Results of Synergy and Sensitivity Testing**

**Figure S1.**

**Figure S1**. (**A**) U251T3 stably expressing mCherry were treated with oHSV (0.05, 0.1 MOI) in 2% FBS DMEM for 48 hours. (**B**) Similarly, GBM6 cells were treated with oHSV )0.05, 0.1, 0.2) in 2% FBS DMEM for 48hours. Cells were collected as a single cell suspension, fixed and permeabilized (R&D Systems), then subjected to spectral flow cytometry measuring viral GFP and Ki-67 staining. Representative dot plots (left) and mean fluorescence intensity plots (right) of replicates are shown.

**Figure S2.**

**Figure S2**. (Left) Representative histological analysis of oHSV-induced proliferation measured by Ki-67 staining in intracranial GBM12 tumor-bearing brain section from mice treated with or without oHSV (Magnification, 20X; scale bar = 200μm).

**Figure S3.**

**Figure S3**. Representative fluorescent microscopy images of MDA-MB-468 mammary fatpad tumors stained for HSV1 (white) and Ki-67 (red), with nuclei counterstained with DAPI (blue) (Magnification = 20X, Scale bar = 200μm). Triplicates shown above of PBS-treated (A) and oHSV-injected (B) tumor sections.

**Figure S4**.

**Figure S4**. Additional Gene Set Enrichment Analyses of our previously published RNA sequencing data of GBM12 (A) and MDA468 (B) treated with oHSV (Q) or without it (N). Heatmaps of z-transformed expression scores of the genes within each gene set that were enriched in the sample are shown to the left of the GSEA enrichment plot. These analyses were performed and graphs prepared using GSEAPy with python.

**Figure S5**.

**Figure S5**. Single channel images of the IF staining depicted in Figure 2B (Magnification 20X; scale bar = 100µm). Fvb/n mice were implanted with the murine breast cancer cell line, DB7, intracranially. 10 days post-implantation, mice were treated with either PBS or 1x10^5^pfu of oHSV and tissues were collected 48 hours post virus injection. Brain sections were stained as described in the Methods using ICP4 and p-IGF1R, with nuclei counterstained with DAPI.

**Figure S6**.

**Figure S6**. Microscope images of Figure 2C are depicted alone with the background signal (secondary only controls) (magnification = 4X, scale bar = 500µm). Athymic nude mice were implanted with GBM12 intracranially. 10 days post-implantation, tumor-bearing mice were treated with either PBS or 1x10^5^ pfu of oHSV. Forty-eight hours post virus injection, brain tissues were collected, fixed in formalin, and then embedded in paraffin.

**Figure S7**.

**Figure S7**. GBM6 and MDA-MB-468 cells were infected with/without oHSV (dose indicated in figure), irradiated with 5 Gy one hour later, and treated with OSI-906 or DMSO solvent control 1 hour after irradiation. Forty-eight hours later, cells were collected as a single cell suspension and stained with the LIVE/DEAD Fixable Near-IR Dead Cell Stain Kit (Invitrogen), then subjected to spectral flow cytometry measuring viral GFP controlled by an immediate early gene promoter and LIVE/Dead staining.

**Figure S8**.

**Figure S8**. **Effect of OSI-906 and RTx on viral kinetics and titers in BC cells.** MDA-MB-468 cells were infected with oHSV (MOI = 0.05), 1 hour later, irradiated with 0, 1, 2, or 5 Gy. One hour after irradiation, cells were treated with DMSO or OSI-906 at 0.05, 0.1, 0.5, 1, or 5μM (*n* = 4/group). Viral propagation and replication were measured every 2 hours for 48 hours utilizing the Cytation 5 live imaging system. Viral GFP count was quantified and graphed as an average of 3 wells per treatment group. Data shown are average counts of GFP-positive cells ± SD over time. Forty-eight hours after virus infection, images were captured, and cells and media were collected for virus titration by standard plaque-forming assay (magnification = 4X, scale bar = 500 μm).

**Figure S9**.

**B.**

**A.**

**C.**

**Figure S9**. **Effect of OSI-906 and RTx on viral kinetics and titers in patient-derived primary GBM cells.** GBM6 cells were infected with oHSV (MOI = 0.05), 1 hour later, irradiated with 0, 2, or 5 Gy. One hour after irradiation, cells were treated with DMSO or OSI-906 at 0.05, 0.1, 0.5, 1, or 5μM (*n* = 4/group). Viral propagation and replication were measured every 2 hours for 48 hours utilizing the Cytation 5 live imaging system. Viral GFP count was quantified and graphed as an average of 3 wells per treatment group. Data shown are average counts of GFP-positive cells ± SD over time. Forty-eight hours after virus infection, images were captured, and cells and media were collected for virus titration by standard plaque-forming assay (magnification = 4X, scale bar = 500 μm).

**Figure S10**.

**Figure S10**. Athymic nude mice were implanted intracranially with GBM12 (left) or GBM28 (right). Ten days after tumor implantation, PBS or 1x10^5^ pfu of oHSV was administered intratumorally, and mice were treated daily with 25mg/kg OSI-906 or an equivalent volume of DMSO by daily oral gavage for 4 weeks.

**Figure S11**.

*

*

*

*

**Figure S11**. **oHSV treatment sensitizes infected GBM cells to IGF1R inhibition, enhancing cytotoxicity.** Cell Viability assay of patient-derived primary GBM cells and U251T3 glioma cell lines treated with or without oHSV and incubated for two hours then treated with DMSO solvent control or the small molecule IGF1R-inhibitor, PPP (0.5μM) for 96 hours (n = 4/group). Cytotoxicity was determined relative to the untreated samples. All data are presented as means ± SD with * p < 0.01.

**Figure S12.**

**Figure S12. oHSV Treatment alone upregulates the Hippo signaling pathway in GBM and MDA-MB-468 *cells in vitro*.** Additional Gene Set Enrichment Analyses of our previously published RNA sequencing data of GBM12 (A) and MDA468 (B) treated with oHSV (Q) or without it (N). Heatmaps of z-transformed expression scores of the genes within each gene set that were enriched in the sample are shown to the left of the GSEA enrichment plot. These analyses were performed and graphs prepared using GSEAPy with python.

**Figure S13**.

**Figure S13**. **Triple Combination Cytotoxicity and Synergy Quantification.** All three cells (MDA-MB-231, GBM28, and GBM6) were plated at 1x10^4^cells/well in 96-well plates. After 24 hours, cells were infected with a serial dilution of oHSV. One hour later, cells were irradiated with 0, 2, or 5 Gy of RTx, followed 1 hour later by treatment with a serial dilution of OSI-906 (DMSO, 0.05, 0.1, 0.5, 1, and 5 uM). MTT cell viability assays were performed 96 hours later. **A.** Dose-response curves for each monotherapy were calculated using % inhibition and log (dose). **B.** ZIP Synergy interaction landscapes for dual therapies are shown as heat maps of % inhibition. Mean ZIP scores are calculated for each dual therapy. **Table S2** shown for a summary of all synergy calculations and sensitivity scores.

**Figure S14**.

**Figure S14**. **Soft Agar Colony Formation Assay.** GBM6 cells were infected with/without 0.1 MOI of oHSV. One hour later, cells were irradiated with 0, 2, or 5 Gy, followed 1 hour later by treatment with DMSO or 5uM OSI-906. At 24 hours post-treatment, single cell suspensions (1x10^4^ cells/well) were embedded in 8% FBS, 0.5X DMEM, 6X Glutamine, and 1.4% agarose in distilled water (DW). Once solidified, a mixture of 0.8% agarose, 10% FBS, and 0.5X DMEM was overlaid on top, and cultures were monitored for colony growth over 3 weeks. Microscopic images were taken from at least three representative fields per well, colony number, shape, and size were quantified using ImageJ. Wells were then fixed with 2% PFA, stained with 0.005% Crystal Violet, rinsed with DW, and imaged. Representative 4X microscope images of colony morphology and density (left), complete macroscopic images are shown (left) and quantified (right) using ColonyArea (Guzmán et al., 2014). Colony staining intensity and percentage area coverage were quantified and plotted.

­­­**Figure S15**.

**Figure S15**. (**A**) The log2 mRNA expression of YAP1 in gliomas (left) and Kaplan-Meir survival curve with median split grouping based on YAP1 expression (right) in male and female patients acquired using the Rembrandt data set. (**B**) The log2 mRNA expression of YAP1 in GBM (left) and Kaplan-Meir survival curve with median split grouping based on YAP1 expression (right) in female patients acquired using the Agilent-4502A Platform TCGA-GBM data set. (**C**) Kaplan-Meir Survival Curves using the TCGA-GBM Database. There is no significant correlation between YAP1 expression and GBM patient survival rate. The addition of male patients removes the survival benefit seen by low-expressing female patients.

**Citations:**

Benjamini, Y., Krieger, A. M., & Yekutieli, D. (2006). Adaptive linear step-up procedures that control the false discovery rate. *Biometrika*, *93*(3), 491-507. <https://doi.org/10.1093/biomet/93.3.491>

Bliss, C. I. (1939). The toxicity of poisons applied jointly 1. *Annals of applied biology*, *26*(3), 585-615.

Franken, N. A. P., Rodermond, H. M., Stap, J., Haveman, J., & Van Bree, C. (2006). Clonogenic assay of cells in vitro. *Nature Protocols*, *1*(5), 2315-2319. <https://doi.org/10.1038/nprot.2006.339>

Guzmán, C., Bagga, M., Kaur, A., Westermarck, J., & Abankwa, D. (2014). ColonyArea: An ImageJ Plugin to Automatically Quantify Colony Formation in Clonogenic Assays. *PloS one*, *9*(3), e92444. <https://doi.org/10.1371/journal.pone.0092444>

Kurozumi, K., Hardcastle, J., Thakur, R., Shroll, J., Nowicki, M., Otsuki, A., Chiocca, E. A., & Kaur, B. (2008). Oncolytic HSV-1 infection of tumors induces angiogenesis and upregulates CYR61. *Molecular Therapy*, *16*(8), 1382-1391.

Loewe, S. (1953). The problem of synergism and antagonism of combined drugs. *Arzneimittel-forschung*, *3*(6), 285-290.

Malyutina, A., Majumder, M. M., Wang, W., Pessia, A., Heckman, C. A., & Tang, J. (2019). Drug combination sensitivity scoring facilitates the discovery of synergistic and efficacious drug combinations in cancer. *PLOS Computational Biology*, *15*(5), e1006752. <https://doi.org/10.1371/journal.pcbi.1006752>

Noh, M. H., Kang, J. M., Miller, A. A., Nguyen, G., Huang, M., Shim, J. S., Bueso-Perez, A. J., Murphy, S. A., Rivera-Caraballo, K. A., Otani, Y., Kim, E., Yoo, S.-H., Yan, Y., Banasavadi-Siddegowda, Y., Nakashima, H., Chiocca, E. A., Kaur, B., Zhao, Z., Lee, T. J., & Yoo, J. Y. (2024). Targeting IGF2 to reprogram the tumor microenvironment for enhanced viro-immunotherapy. *Neuro-Oncology*. <https://doi.org/10.1093/neuonc/noae105>

Wakimoto, H., Fulci, G., Tyminski, E., & Chiocca, E. A. (2004). Altered expression of antiviral cytokine mRNAs associated with cyclophosphamide's enhancement of viral oncolysis. *Gene Ther*, *11*(2), 214-223. <https://doi.org/10.1038/sj.gt.3302143>

Yadav, B., Wennerberg, K., Aittokallio, T., & Tang, J. (2015). Searching for Drug Synergy in Complex Dose–Response Landscapes Using an Interaction Potency Model. *Computational and Structural Biotechnology Journal*, *13*, 504-513. <https://doi.org/10.1016/j.csbj.2015.09.001>

Zheng, S., Wang, W., Aldahdooh, J., Malyutina, A., Shadbahr, T., Tanoli, Z., Pessia, A., & Tang, J. (2022). SynergyFinder Plus: Toward Better Interpretation and Annotation of Drug Combination Screening Datasets. *Genomics, Proteomics &amp; Bioinformatics*, *20*(3), 587-596. <https://doi.org/10.1016/j.gpb.2022.01.004>
